# Optimizing fountain codes for DNA data storage

**DOI:** 10.1016/j.csbj.2024.10.038

**Published:** 2024-10-26

**Authors:** Peter Michael Schwarz, Bernd Freisleben

**Affiliations:** Department of Mathematics and Computer Science, University of Marburg, Hans-Meerwein-Str. 6, D-35043, Marburg, Germany

**Keywords:** DNA, DNA storage, Optimization, Fountain codes

## Abstract

Fountain codes, originally developed for reliable multicasting in communication networks, are effectively applied in various data transmission and storage systems. Their recent use in DNA data storage systems has unique challenges, since the DNA storage channel deviates from the traditional Gaussian white noise erasure model considered in communication networks and has several restrictions as well as special properties. Thus, optimizing fountain codes to address these challenges promises to improve their overall usability in DNA data storage systems. In this article, we present several methods for optimizing fountain codes for DNA data storage. Apart from generally applicable optimizations for fountain codes, we propose optimization algorithms to create tailored distribution functions of fountain codes, which is novel in the context of DNA data storage. We evaluate the proposed methods in terms of various metrics related to the DNA storage channel. Our evaluation shows that optimizing fountain codes for DNA data storage can significantly enhance the reliability and capacity of DNA data storage systems. The developed methods represent a step forward in harnessing the full potential of fountain codes for DNA-based data storage applications. The new coding schemes and all developed methods are available under a free and open-source software license.

## Graphical abstract

**Figure:**
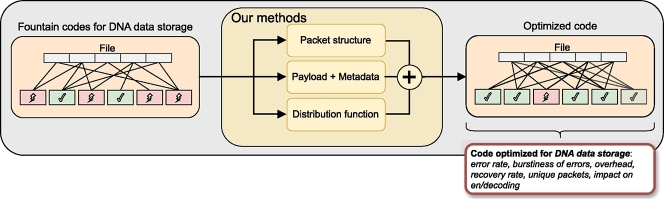

## 1 Introduction

The global demand for data storage is growing significantly, with estimates projecting the need to store 175 zettabytes (1 zettabyte = 10^21^ bytes) by 2025 [1]. Traditional storage media such as hard drives, magnetic tapes, and optical discs have limitations in terms of storage density, durability, resource usage, and energy consumption [2]. DNA storage is a promising alternative, with a theoretical storage density of up to 455 exabytes per gram [3], crucially surpassing conventional storage media. Properly stored, DNA can remain stable for thousands of years [4], [5], ensuring long-term data preservation. Compared to hard drives with typical lifetimes of 3–10 years [6] and capacities around 22 TB, and magnetic tapes with lifetimes of around 30 years [7] and densities of up to 580 TB per tape (317 GB/in^2^) [8], DNA clearly outperforms conventional storage technologies in terms of both density and longevity. Moreover, DNA storage addresses environmental issues associated with conventional storage, such as the use of rare earths or other materials that raise environmental concerns during extraction and processing. After all, it is expected that traditional storage systems will not be able to keep up with the ever-growing amount of data created worldwide, leading to the inability to store the entirety of created data [9].

DNA's relevance to humanity inherently ensures the continued development and improvement of DNA reading, amplification, and writing technologies. Advancements in DNA synthesis, polymerase chain reaction (PCR), and DNA sequencing directly improve DNA storage without the need for dedicated read/write technologies required for conventional storage systems like solid-state drives. Potential research in the area of in-vivo DNA storage opens possibilities for self-repairing and reproducing data storage, which further enhances the reliability of DNA storage systems. With its energy efficiency, high density, long-term stability, ease of replication, biological nature, and potential for cheap duplication, DNA has emerged as a highly promising medium for digital data storage in recent years.

Since DNA molecules consist of a sequence of the four nucleotides Adenine (A), Guanine (G), Cytosine (C), and Thymine (T), binary data has to be translated into this quaternary representation. To enhance the resilience of data decoding in the presence of errors and biological constraints associated with DNA synthesis, storage, and sequencing technologies, it is common to add up to 30% redundancy in the form of error-correcting codes, which work well when the error rate is low, typically below 5% [10], [11], [12]. The encoded data is then “written” using DNA synthesis, a method that can create de-novo sequences. The resulting and typically rather short fragments, commonly called oligonucleotides (short: oligos), range from 40 to approximately 300 base pairs (bp). For the actual storage of these oligos, various approaches including in-vivo storage exist, but most commonly, the synthesized sequences are stored in-vitro. To “read” the data from the sequences, DNA sequencing technologies are used. These produce text files that include the order of the different nucleotides (nt) as well as information about the sequencing accuracy.

Typical errors are random mutations, insertions, and deletions during the storage process, and the most prominent biological constraints any DNA sequence to be stored has to adhere to are related to: (1) run length, (2) GC content, and (3) homopolymers. The first constraint limits the length of each DNA sequence to be synthesized to approximately 200-300 nt [13], since the cost of synthesizing long strands of DNA significantly increases due to declining stability, which in turn decreases overall reliability. Thus, to encode large digital files, the use of indexing structures is required. The second constraint limits the GC content, i.e., the percentage of G and C nucleotides compared to the overall length. Since the GC content influences the melting temperature of the DNA and thus its stability during synthesis, PCR, and sequencing, this restriction is often applied to each DNA sequence as well as to windows inside each DNA sequence. The third constraint limits the number of nucleotides allowed to appear consecutively in a sequence to 3-4 equal nucleotides [14], since larger numbers might lead to various problems such as slippage during PCR. Additional constraints, such as undesired DNA subsequences and the avoidance of sequences with a high likelihood of secondary structures, may be applied if required.

Error correction and constraint satisfaction in DNA data storage can be addressed by fountain codes [15], [16], [17], [18]. Fountain codes [19] have proven to be highly versatile and beneficially applicable in various digital data transmission (and storage) scenarios, with an information rate that theoretically allows encoding information near the channel capacity. Basically, fountain codes are used to break the data to be encoded into chunks, from which many possible packets are generated. Thus, fountain codes offer the potential to generate infinitely many encoded symbols for binary data to be reliably transmitted between one sender and one or multiple receivers over well-defined erasure channels (with practical limitations). Only a small subset of (1+ϵ)⋅n (where *n* is the number of chunks a file to be encoded has been split into and *ϵ* is the required overhead) encoded symbols needs to be received to fully reconstruct the original data. The order of symbols or which particular symbols are received is not relevant, as long as enough symbols arrive.

In practice, fountain codes work in the following manner. First, the file to encode is split into equally sized chunks. Then, a distribution function is sampled to assign a degree to each packet. These packets are filled with pseudo-randomly selected chunks through XOR operations based on their degrees, and then transmitted through an erasure channel to the recipient. Upon obtaining a sufficient subset of these transmitted packets, the receiver utilizes the received packets along with information about the contained chunks to reconstruct the original data. For successful reconstruction, the receiver needs either a list of chunks integrated into a given packet (e.g., as part of the transmission) or knowledge about the distribution function and the seed that initialized the random number generator used during encoding. Given that the distribution function describes the proportion of packets for each degree, a modification has implications regarding the en- and decoding complexity (e.g., a higher average degree increases the number of XOR operations to perform), the expected overhead required for decoding, the resilience against erasures, the expected entropy of the encoded packets (e.g., when encoding a low entropy file, packets with a low degree are more likely to produce a low entropy payload), as well as the number of duplicate packets to expect over the range of possible seeds.

In DNA storage scenarios, fountain codes treat synthesized DNA fragments as packets in a data stream, using an inner error-correcting code to safeguard each DNA fragment. If the inner code detects errors that it cannot correct, such as insertion and deletion errors (indels) or excessive substitutions, the individual DNA fragment is treated as an erasure. Erasures can be reconstructed from other DNA fragments using an outer fountain code.

Compared to other codes used for DNA data storage, fountain codes have several beneficial properties. First, by generating numerous packets for a given input, they can achieve a code rate near the channel capacity by finding and filtering out rule-violating packets without costly translation or bit-stuffing techniques. Additionally, their unordered nature perfectly matches the DNA medium, which allows the data to be stored without additional indexing schemes. Thus, the seed used to encode a packet is sufficient to explicitly define the content stored in the packet. Furthermore, the dropout or failure to read sequences matches the ability of fountain codes to decode from any sufficiently large subset of received packets. Compared to Reed-Solomon (RS) codes [20], fountain codes can guarantee the adherence to specific rules. Since Reed-Solomon codes typically operate in a systematic manner (i.e., they append recovery information after the actual payload) and do not allow the creation of additional packets for a given input, the sole use of Reed-Solomon codes for encoding binary information is not feasible for the DNA storage use case and may only be efficiently usable as a part of a concatenation scheme. This applies to all strictly systematic codes and especially for codes incapable of generating infinitely many packets from a finite set of source symbols. While there are codes, such as the one developed by Grass et al. [4], that use Reed-Solomon codes and can avoid homopolymers (e.g., using Galois field translations) at the expense of significantly reducing the possible information rate, they are typically based on a fixed set of rules the generated sequences have to adhere to. Modifying these rules would require a new or significantly modified coding scheme. For example, the code developed by Grass et al. cannot reliably enforce a GC content for generated packets or avoid arbitrary sets of subsequences. Using the configuration defined by Grass et al., the developed code has an information rate of 1.187 with a net information density of 1.14, as calculated by Erlich and Zielinski [15]. In comparison, fountain codes as used in *DNA Fountain* [15], *NOREC4DNA* [16] or *DNA-Aeon* [21] can achieve comparable rule abidance including homopolymers, GC content, and the additional option to filter out sequences matching arbitrary rules. This allows coding schemes based on fountain codes to easily adapt to new or modified restrictions as they commonly arise in the diverse and quickly changing field of synthetic biology. Given a GC content of 45%-55% and a maximum homopolymer length of 3 over a sequence length of 150, Erlich and Zielinski calculated a coding potential of 1.98 bits/nt. By adding the costs of an index to each sequence, this rate would lower to 1.84 bits/nt, assuming a 13 nt long index for each 150 nt sequence. When adding redundancy against dropouts or errors, Erlich and Zielinski state that the reduction in capacity is equivalent to the expected dropout rate [15]. Comparing this to the code of Grass et al., fountain code schemes could protect against a dropout rate of 38% while still maintaining a higher information rate. Since this calculation is independent of a particular fountain code, it applies to both the method used in DNA Fountain [15] and our implementation using a Raptor code [16], [22]. Considering the overhead required for reliable decoding, DNA Fountain with its underlying LT code and robust soliton distribution requires a significantly higher overhead than the modern Raptor codes which typically require an overhead of 1–2 packets for a 99.99% probability of a successful decoding [23], [24]. To summarize, fountain code schemes are capable of trading computational complexity (in the form of computing additional packets) for reduced overhead, since no additional coding is required to adhere to any restrictions required for DNA storage. This property holds for codes that can produce a variable and potentially infinite number of different packets for a single input file. Since DNA storage does not require live transmission of data, all possible packets can be pre-computed. This allows the selection of the best packets (e.g., minimum error probability, overhead required, and highest recovery rate for partial recovery), which makes fountain codes an ideal candidate for developing encoding schemes for DNA storage.

From the perspective of improving codes for DNA data storage, current research focuses on enhancing the storage density [21], finding new methods to store specific information (e.g. databases or images) [25], and novel ways of storing information using DNA, such as DNA origami [26]. Furthermore, there are several works focusing on DNA coding schemes to satisfy specific assumptions and restrictions [15], [16], [21]. Apart from the initial idea of using conventional fountain code approaches for DNA data storage by Erlich and Zielinski [15], previous research on fountain code optimization has focused on improving performance for traditional data transmission scenarios [27], [28] that do not account for the unique constraints and error models involved in the DNA data storage process. Furthermore, there are recent works that focus on providing general design considerations for long-term data storage using DNA [29], but they do not focus on fountain codes and their optimization for DNA data storage. However, using fountain codes for DNA data storage has unique challenges that were previously not considered in any work related to the optimization of fountain codes:

• The underlying DNA storage channel does not resemble a conventional erasure channel, but is based on an error model that is partially dependent on the stored content, and the introduced errors are not equally distributed. While an erasure channel can, to a certain degree, be simulated by introducing error detecting checksums, current distribution functions for fountain codes are primarily optimized for binary symmetric transmission channels. In contrast, the DNA storage model operates in the quaternary system consisting of four DNA nucleotides. The error probability of a nucleotide depends partially on the structural characteristics of the stored data.
• DNA data storage is fundamentally different from traditional data transmission scenarios, leading to a distinct set of optimization goals. For instance, in DNA data storage, the mentioned restrictions must be addressed.
• The constraints regarding time and space complexity for encoding and decoding in DNA data storage systems are not as critical as for conventional data storage and data transmission systems. The reason is that DNA data storage is inherently limited by DNA synthesis, PCR, storage conditions, and DNA sequencing, which are constrained by both the current technology and the biological processes themselves.
• The synthesis of long DNA sequences is still costly [30], [9]. Since the costs of biological processes are significantly higher than the typical computational costs for en- and decoding, more sophisticated and computationally expensive codes can be used to satisfy the biological restrictions and thus reduce the overall storage costs.

Despite these challenges, the high storage density and long service life of DNA make it a promising solution for long-term data storage, particularly for archival data that is not accessed frequently [31], [32], [33].

The requirements and restrictions of the DNA storage channel significantly differ from the previous optimization targets of minimal overhead and reduced computational cost for the transmission over conventional and well-defined transmission channels as highlighted by Chen et al. [28], who used a multiobjective evolutionary algorithm. Likewise, there are various works offering mathematical approaches for optimizing the distribution function of fountain codes in the conventional erasure channel setting [34], [35], [36], but these approaches are not feasible for encoding data for storage in DNA. Furthermore, the typical process of using fountain codes in DNA data storage neither involves methods to reduce the impact of the seed nor the impact of the actual payload to encode with respect to constraint violations.

Since the optimization of fountain codes for DNA data storage is a novel research area, several research questions arise: (1) How can fountain codes be optimized to address the unique challenges and constraints of using DNA as a data storage medium? (2) How does content and metadata to be encoded influence the probability of generating rule-abiding packets? (3) What are effective methods to reduce the probability of generating DNA sequences that violate biological constraints, such as homopolymer runs and undesirable GC content, when using fountain codes? (4) How does the degree distribution function of fountain codes impact key metrics such as encoding efficiency, error correction capability, and storage density when applied to DNA storage? (5) How should the use of a non-standard distribution function be stored to allow a resilient and low overhead decoding?

In this article, we address these research questions by optimizing fountain codes to improve their overall usability in DNA data storage systems, producing a novel coding scheme that is more scalable and less error-prone than existing fountain code schemes for storing digital information in DNA. Apart from generally applicable optimizations of fountain codes, we propose optimization algorithms to create tailored distribution functions for fountain codes, which is novel in the context of DNA data storage. We evaluate the proposed methods in terms of various metrics related to the DNA storage channel. Our focus is on improving the encoding efficiency and error correction capabilities of fountain codes for DNA data storage, with the ultimate goal of improving the reliability and capacity of DNA data storage systems.

Our approach of improving fountain codes by increasing the entropy of created packets and optimizing the distribution function towards specific metrics important for DNA data storage is entirely novel.

Our contributions are as follows:

• We present novel optimization methods for fountain codes in DNA data storage, including seed spacing and payload masking, to decouple structure and content of the payload from constraint violations. These methods significantly improve usability and efficiency of DNA data storage schemes based on fountain coding approaches.
• We propose novel optimization algorithms to create distribution functions specifically for DNA data storage, improving reliability and increasing potential code rates by focusing on the defined optimization goals.
• We evaluate the proposed methods using metrics relevant for the DNA data storage channel. Based on our findings, we present best practices for developing DNA data coding schemes using fountain codes and introduce a novel code that outperforms comparable approaches in terms of required overhead, error rates, storage density, and recovery capabilities.

## 2 Materials and methods

### 2.1 Optimization objectives

The ideal requirements of fountain codes, such as LT [37], Online [38] or Raptor [39], for data transmission in communication systems, are as follows [19], [39]:

1. A sender should be able to generate as many encoded symbols as required.
2. In the majority of scenarios, a receiver should have the ability to decode an exact copy of the original data from any subset of *k* encoded symbols. This should be possible regardless of which subset was received and whether it was received from one or multiple senders.
3. The computational time required for both encoding and decoding should be linear, proportional to the size of the original source block size used by the sender for transmission.

In the absence of a sender and receiver, we can adapt these requirements to better align them with the requirements of DNA data storage:

1. The encoding process should generate all encoding symbols within the designated seed space and reduce the number of duplicate sequences and data size to encode in DNA.
2. In most scenarios, it should be possible to decode an exact copy of the original data from any subset of *k* encoded symbols, regardless of the subset that was stored in DNA.
3. While maintaining a reasonable computational efficiency is important, the primary focus is to ensure reliable recovery of the data stored in DNA. Since DNA storage does not have real-time access restrictions, an increased computational complexity is tolerable.
4. A minimum of *k* (n≤k) encoded symbols must adhere to the restrictions of the DNA storage channel.
5. If an exact decoding is not possible for a subset $k^{\prime },k\subseteq k^{\prime }$, the number of decoded source blocks should be maximized. Compared to a transmission-based approach, no additional packets may be received, i.e., it is critical to be able to retrieve as much of the stored file as possible.

These requirements lead to several objectives for fountain codes in DNA data storage:

1. The general optimization objectives that all fountain codes (including fountain codes for DNA data storage) should satisfy are as follows:
  (a) **MinAvg**: find a distribution function that (on average) minimizes the packets (E[T]) required to successfully decode the data, where *T* is the number of received packets required for a successful decoding.
  (b) **MaxPr**: find a distribution function that maximizes the probability of decoding a message with exactly *n* (or n+k,k>0) packets, where *n* is the number of chunks (i.e., the original message has been split into $overhead=\frac{E[T]-n}{n}$ packets).
2. Apart from these general optimization objectives, there are additional objectives for fountain codes to be used in DNA data storage systems, which we define as follows:
  (a) **MaxDecode**: maximize the number of decoded blocks for a partially solved equation system represented by fountain codes.
  (b) **MinError**: minimize the average error estimation per packet.
  (c) **MaxUniquePackets**: maximize the number of (unique) packets that can be generated. While a low average degree is beneficial for decoding, it might be required for a DNA data storage channel that a large amount of unique packets can be generated to filter sequences that do not satisfy the biological restrictions. Since the number of unique packets that can be generated for each degree *deg* is defined by $\left(\begin{array}{c} n\\ deg \end{array}\right)$, we can directly conclude that increasing the probability of a higher *deg* will result in more unique packets available. This implies that fewer packets with different seeds will result in the same encoded payload (by sampling the same chunks for XOR).
  (d) **MaxCleanDegLen**: maximize the number of packets with an error estimation <1.0
  (e) **MinAvgErrSmallerOne**: minimize the error for packets with an error <1.0
  (f) **MinBurstError**: minimize the burstiness of rule-violating packets.

**MinError**, **MaxUniquePackets**, **MaxCleanDegLen**, and **MinAvgErrSmallerOne** can be calculated for each distribution by creating all packets for the selected seed space, but this approach is not feasible for the remaining objectives. In the context of fountain codes, particularly when applied to DNA data storage, there is no predetermined transmission order. Consequently, it is not possible to assume a specific receiving order or make statistical claims regarding the packets already received. Therefore, to accurately compute **MinAvg**, **MaxPr**, and **MaxDecode**, we have to decode all $\sum_{\epsilon }\left(\begin{array}{c} x\\ n+\epsilon \end{array}\right)$ possible combinations of packets, where $x=2^{l}$ is the total number of all possible packets limited by the number of bytes *l* of the seed. Given that this exhaustive approach is impractical, we use a sampling method as outlined in the following steps:

1. **MinAvg**: This metric is defined as the average number of packets required to decode the original file successfully. To avoid outliers, the average of *i* random repetitions is used.
2. **MaxPr**: This metric is also calculated over *i* iterations. However, in this case, exactly *n* packets are inserted into the decoder in each iteration. **MaxPr** is then defined as the average success rate of the decoding process over the *i* repetitions.
3. **MaxDecode**: For this metric, exactly *n* packets are inserted into the decoder, after which the number of correctly decoded chunks is recorded (equal to *n* if decoding is successful). To avoid outliers, the average of *i* random sets of *n* packets is used.

Compared to the use of fountain codes for data transmission in communication systems, DNA data storage does not require real-time encoding and decoding. Moreover, the store-once architecture of DNA prohibits the creation of additional packets, and the DNA storage medium is susceptible to a series of errors and erasures. However, pre-computation is possible, enabling an encoder to compute all possible packets *P* and subsequently select the optimal subset s⊆P to store in DNA. In this context, “optimal” can be defined using the following criteria: (1) minimal error probability, (2) minimal required overhead for each unique subset (thus ensuring successful decoding independently of which encoded packet(s) were erroneous) or (3) maximum distance between all encoded sequences.

### 2.2 Seed masking

**Fig. 1:**
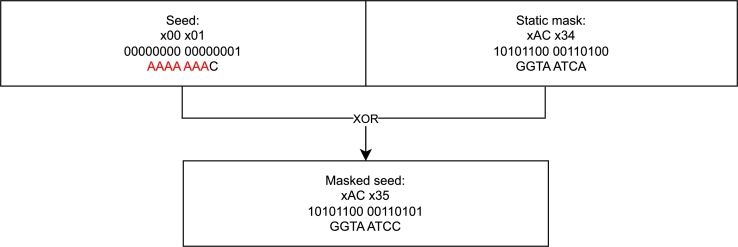
Seed masking. The two byte seed that would produce a homopolymer is combined with a fixed seed using the XOR function. The resulting seed does not produce a homopolymer. This supports the iterative creation of packets, spacing out the impact of homopolymers in the seed. When generating all possible packets, the number of homopolymer-producing seeds does not change using this method.

The first method we present to optimize fountain codes for DNA data storage purposes is XOR masking of the seed using a known value of identical length, as shown in Fig. 1.

This method was used in NOREC4DNA [16] to decrease the burstiness of rule-violating packets by reducing the likelihood of homopolymer formation or unfavorable GC content. Here, the primary goal of this method is to introduce randomness into the seed's impact during the iterative packet generation process. Thus, we can create all packets starting from seed 0, ensuring that the leading zeros do not contradict any channel restrictions. Consequently, this method accelerates the real-time creation of a predetermined quantity of packets in the case of strictly iterative packet creation. The mask should be chosen such that it minimizes the burstiness of rule violations resulting from the seed. For this purpose, the static mask “xAC x34” used in Figs. 1 is a suitable candidate, especially for smaller seeds, since it avoids leading homopolymers. However, since the used mask only influences the order in which homopolymers and unfavorable GC content regions occur in the seed region, other masks can be used. When choosing a different mask, two main considerations have to be considered: (1) what is the largest range of seeds that does not lead to rule-violating subsequences and (2) how can the used mask be transmitted to the receiver. For (1), the range of seeds typically starts with 0 and ranges up to an arbitrary number deemed suitable for the given experiment, which is usually slightly larger than the number of chunks. Selecting a mask then involves either manually finding a mask that produces a minimal number of seeds that result in rule violations, or computing this mask automatically. To automatically compute a mask, one could analyze the most common regions of the seed that produce rule violations, such as homopolymer runs over the previously selected seed range. Given this information, the mask should be set such that these regions do not lead to rule-violations above the average for all positions of the seed. While such an exhaustive search for an ideal mask may be computationally expensive and has to be performed for any seed range of interest, a typical approach is the use of approximations breaking up any long repeats of leading zeros. For (2), there are various approaches. The most straightforward approach is the additional storage in DNA as a single packet, but this has the following drawbacks. It reduces the storage density and thus increases the cost, requires the mask to be free of rule-violations itself, and results in a fatal data loss if the sequence suffers from errors or a dropout. A different possibility is to use a finite set of pre-defined masks and decode the stored content once for each of the masks. This approach thus does not require any additional overhead and also does not expose a higher risk of a decoding failure due to a single dropout or error. However, if all decoding runs are not successful, this approach would further complicate a generally computationally more expensive recovery process. Lastly, a single mask could be used that is optimized for a reasonable range of seeds, resulting in improved performance for the average of small or medium-sized experiments. An illustration of the effect seed masking may have for consecutive seeds can be found in the supplemental material. It is important to note that this approach effectively shuffles the seed space, leading to an improved encoding speed only if not all possible packets are generated. If all packets for a given seed range must be generated, the overall number of packets influenced by rule-violating (masked) seeds is equal to not using such a mask. Thus, this method is primarily applicable to achieve the **MinBurstError** objective.

### 2.3 Seed spacing

To actually decouple the structure of the seed from the likelihood of a rule violation, we present the more promising method of seed spacing. This method involves distributing the seed throughout a packet by interleaving it with the actual payload. Using this method, we can specify seed spacing by defining the number of payload bases (i.e., nucleotides) positioned between each base of the seed. This not only diminishes the influence of rule-violating seeds with fixed length, but also accommodates the use of larger seeds, which would typically introduce a substantial number of rule-violating sequences. This method allows the utilization of the entire range of a 4-byte seed space, thereby enabling the direct storage of files up to approximately 160 GB without the need for sub-packets (assuming 40 bytes per packet).

**Fig. 2:**
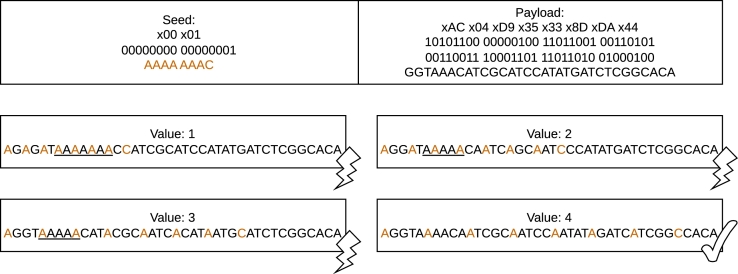
Seed spacing with different interleaving values for a specific payload. Assuming a maximum allowed homopolymer length of 4, in the absence of interleaving, the reason for the rule violation is the seed itself. This method can be used both per byte and per base (i.e., nucleotide), offering fine-grained control over the interleaving process.

Fig. 2 presents the method of seed spacing for various interleaving values. When no interleaving is applied (spacing =0), the translation to a DNA sequence produces a homopolymer of length 7. However, as we start introducing a one-base spacing, we observe that the formation of the homopolymer is no longer solely attributed to the seed; it is now influenced by the actual payload. Ultimately, with a spacing of 4 bases, the content of this generated packet no longer violates the homopolymer constraint, satisfying the constraint of the presented example. By increasing the randomness of the generated payload, this method reduces the overall number of rule-violating sequences by preventing the creation of packets with homopolymers introduced by the seed.

Compared to the methods used by Erlich and Zielinski [15], seed spacing can minimize the influence of the seed on the overall error probability of the generated packets. While DNA Fountain [15] with its fixed size seed of 4 bytes and rule-based system, can avoid homopolymer runs and maintain GC content, homopolymer runs of length 3 or more inside the seed region reduce the number of possible seeds by 2,284,930,588 (643,053,460 limiting homopolymer runs to be 3 or less). Thus, 53.2% (15% when allowing homopolymer runs of 3) of all possible packets created using DNA Fountain produce packets not suitable for storage in DNA before even filtering out sequences that contain any homopolymers inside the actual payload or expose an unfavorable GC content.

In a typical setting of a 2 byte Reed-Solomon code, a 4 byte seed and a maximum sequence length of 300 nt per packet, each chunk would have to be 69 bytes long. With this information, the maximum file size that DNA Fountain could ideally handle would be $2^{32}\cdot 69\cdot (1-0.532)=138,693,083,922$ bytes, which is equivalent to approximately 129.17 GB (234.6 GB when allowing homopolymers of length 3 or less). If additional overhead, duplicate encoded payloads due to the unsuitable distribution function and rule-violating payloads are assumed, the actual maximum file size usable with DNA Fountain is expected to be significantly smaller. By spacing the seed over the encoded payload, the impact of the seed on any rules is minimized, making rule violations only dependent on the encoded content.

The relevance of seed spacing in experiments involving longer seeds of 4 or more bytes increases, since the number of homopolymer-producing seeds is higher in such scenarios. Depending on the seed length and the interleaving step size, this method may also influence the GC content per window. Given its payload-altering nature, this method can be used to optimize fountain codes to achieve the following objectives: **MinError**, **MaxCleanDegLen**, and **MinAvgErrSmallerOne**.

### 2.4 Increasing entropy by compressing or encrypting the input

After having minimized the impact of the seed on the likelihood of a rule violation, we now attempt to minimize the impact of the actual payload on rule violations by increasing the entropy of the encoded sequences.

The maximum possible entropy for an alphabet *A* is determined by:

$$H_{max}(F)=-\sum_{a\in A}p_{F}(a)\log_{2}(p_{F}(a))=\log_{2}(n)\text{where }A=\{a_{1},\ldots ,a_{n}\}\text{ and }\forall a\in A:p_{F}(a)=\frac{1}{n}$$

For bytes, this results in a theoretical maximum entropy of 8. However, in the case of DNA, the maximum value is 2. Since the used error model operates on the quaternary DNA representation, we use the quaternary representation for the entropy calculation in the following sections.

A straightforward method to increase the entropy while reducing the volume of data to be stored is to compress the raw input. With the reduced number of total packets, this subsequently increases the possible redundancy for a fixed seed length. It also decreases the average error prediction by reducing the number of consecutive bytes in the raw input, as well as (to a certain degree) equalizing the input bytes, thereby promoting a more favorable GC content. This, in turn, increases the likelihood of generating rule-compliant packets.

An alternative method is data encryption to further increase the entropy of the input to be encoded, which in turn increases the probability of generating rule-compliant packets. However, since this method does not decrease the length of the input, the benefit of a decreased number of input chunks is not available.

Both methods have drawbacks in the event of partial data recovery. If full recovery of the encoded data is not possible, a compressed or encrypted payload may not be (partially) recoverable, potentially resulting in complete data loss. Similarly to seed spacing, these two methods can contribute to the achievement of the following objectives: **MinError**, **MaxCleanDegLen**, and **MinAvgErrSmallerOne**. To a certain extent, these two methods may also help to achieve the **MaxUniquePackets** objective, since a reduction of *n* increases the number of packets available for combining chunks to higher degree packets.

### 2.5 Payload masking

Since compression and encryption modify the raw data to be encoded, which should be avoided for recovery and general usability, a different method for generating more rule-compliant packets is payload masking, i.e., the use of XOR to introduce equally distributed random sequences into the payload. In this method, a uniformly distributed random sequence $s\in X^{n},X∼Ber_{\frac{1}{2}}$ (Bernoulli distribution) with the same length *n* as the payload is added to the encoded payload using the XOR operation (in the finite field $F_{2}$). Since this method is transparent to both the encoder and decoder as long as the added sequence is known, it does not impact the recovery potential in the event of an unsuccessful decoding. To eliminate the need for transmitting the used sequence out of band and to avoid relying on a potentially suboptimal single sequence, we propose to utilize the seed to generate a unique sequence from $X^{n}$ for each packet. When combined with the interleaving of the seed, this method further increases the entropy of the file, creating a more homogeneous distribution of the four bases. An illustration of this method can be found in the supplemental material. Like the previous methods, modifications of the input, including source coding steps, can be used to achieve the objectives **MinError**, **MaxCleanDegLen**, **MinAvgErrSmallerOne**, and **MaxUniquePackets**. Additionally, by significantly decoupling the file's content and its chunks from the error probabilities, this method can contribute to **MinBurstError**, particularly in the case of systematic packet creation, which is used in certain fountain code variants to improve transmission and decoding efficiency.

### 2.6 Optimizing the distribution function

After addressing problems regarding the structure of the generated packets, we will now focus on the design and construction of fountain codes and the packets they produce. The distribution function of a fountain code has a significant impact with respect to its usability, required overhead, and average entropy of the generated packets. Existing distribution functions for fountain codes primarily focus on minimizing the overhead of on-the-fly data transmission (i.e., **MinAvg**, **MaxPr**), while also offering a near-linear complexity. However, this approach tends to favor low degrees, which, in turn, limits the number of possible unique packet payloads (**MaxUniquePackets**) for a given seed space and leads to more rule violations. For example, a distribution function that heavily favors packets of degree 1 will generate many duplicate payloads. This reduces the overall code efficiency while producing a higher percentage of rule-violating packets for low entropy input and also leads to suboptimal decoding properties. In this section, we present a novel approach to address this challenge, taking into account the unique constraints of DNA storage systems.

**Fig. 3:**
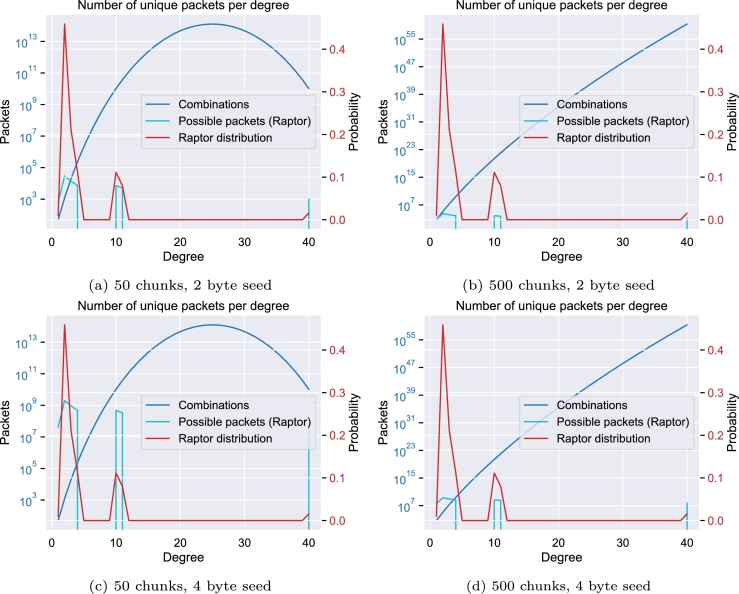
The Raptor distribution function [39] (red), the maximal possible combinations for each degree (blue) as well as the number of possible packets based on the Raptor distribution (light blue) for various numbers of chunks and seed sizes.

Fig. 3 shows the influence of the Raptor distribution function [39] on the possible combinations for each degree *d* regarding the number of chunks *n*, calculated as $p_{d}=\left(\begin{array}{c} n\\ d \end{array}\right)$. In the case of a limited number of chunks and a seed length of 2 bytes, as demonstrated in Fig. 3a, the creation of packets for degrees 1 and 2 (light blue line) exceeds the theoretical maximum for these degrees (blue line). For example, with 50 chunks and a 2-byte seed, there are only $\left(\begin{array}{c} 50\\ 1 \end{array}\right)=50$ unique degree-1 packets and $\left(\begin{array}{c} 50\\ 2 \end{array}\right)=1225$ unique degree-2 packets possible. As this limit is approached, the probability of producing previously unseen packets decreases. Once these limits are reached, any additional packets will necessarily be duplicates, differing only in their seed values. Fig. 3b illustrates that increasing the number of chunks can mitigate this issue.

Evaluations of the robust soliton distribution as used by Erlich and Zielinski [15] can be found in the supplemental material. Since this distribution heavily uses lower degrees, it shows an equally unfavorable behavior. A small number of possible combinations leads to a problem in which a significant number of the created packets have a non-unique payload.

However, in scenarios that involve larger files or constraints on rule-compliant packets, increasing the seed size may be required. Figs. 3c and 3d reveal that the default distribution function may generate a significant number of duplicate packets. The random choice of the packets to combine might even further increase the number of non-unique packets. Our supplementary material includes additional figures for different values of the number of chunks.

To illustrate the impact of the high probability of low-degree packets, we calculate the expected number of required packets to retrieve all unique combinations for a given number of chunks and the expected number of unique packets after *n* generated packets. Assuming n=100 chunks, k=2 as the selected degree, l=2 as the seed length in bytes, and $\left(\begin{array}{c} n\\ k \end{array}\right)=4,950$ unique and equally likely combinations, as well as $N=2^{l⁎8}⁎\text{ Raptor}\_\text{dist}[k]\approx 30,084$ packets expected to be generated for degree 2, we can use the concept of the coupon collector problem [40], [41] to predict the number of packets required to generate all possible unique packets. The coupon collector's problem models the process of collecting a set of distinct objects (coupons) through random sampling with replacement. In our context, the unique packet payloads represent the coupons, and the packet generation process is analogous to the sampling. By applying the results of the coupon collector's problem, we can estimate the expected number of packets that need to be generated to obtain all unique payloads for a given degree.

Thus, we would need to generate $E(N)=n\cdot \sum_{k=1}^{N}(\frac{1}{k})$ packets that can be approximated using E(N)≈4,950⋅(ln⁡(4,950)+0.57721)≈44,968 where 0.57721 is Euler's constant. Therefore, we would need 44,968 packets of degree 2 to ensure that all unique packets were generated, which in turn would mean that approximately 44,968−4,950=40,018 of the generated packets would be duplicates. Calculating the expected number of unique packets after 30,084 packets of degree 2, using the approximation $n\cdot (1-{\left(1-\frac{1}{n}\right)}^{N})$, with n=4,950 and N=30,084, we can expect approximately 4,938 unique packets. Thus, 4,950−4,938=12 distinct packets would still be missing. It is worth noting that the intermediate packets created for the Raptor code are not included in this calculation, but since these packets can be considered to be a linear combination of the source chunks, including them would reduce the expected number of unique packets even further.

Considering the specified constraints and the impracticality of applying the default distribution function to the DNA storage use case, we present a tailored version of the distribution function designed explicitly for the DNA storage channel.

To enhance the likelihood of finding such an optimal solution, we use established optimization techniques to improve the default distribution function for Raptor fountain codes [22], which include our own implementations of well-known methods such as evolutionary optimization and differential evolution [42]. We also implemented a (stochastic) gradient descent optimization method, but it did not produce stable results for the set of hyperparameters used. Thus, we focus on evolutionary optimization methods, since they were proven to be effective for handling the complex search space and multi-objective optimization required for distribution function optimization [28]. Furthermore, as population-based methods, they are well suited for being executed in parallel, thus improving computation time. Since the impact of the different objectives, as elaborated in Section 2.1, might depend on the specific use case, we introduce a set of customizable parameters to create a tailored distribution function. The optimization process can be summarized as the minimization of the following synthetic error value, which is also commonly referred to as *fitness function*, for each generated distribution function:

(1) $$\begin{array}{cc} s= & f_{\mathrm{overhead}}⁎\mathrm{overhead}+f_{\mathrm{avg}\_\mathrm{err}}⁎\mathrm{avg}\_\mathrm{err}\\ & +f_{\mathrm{clean}\_\mathrm{avg}\_\mathrm{err}}⁎\mathrm{clean}\_\mathrm{avg}\_\mathrm{err}\\ & +f_{\mathrm{non}\_\mathrm{unique}\_\mathrm{packets}}⁎\mathrm{non}\_\mathrm{unique}\_\mathrm{packets}\\ & +f_{\mathrm{unrecovered}\_\mathrm{packets}}⁎\mathrm{unrecovered}\_\mathrm{packets} \end{array}$$

Here, $f_{\mathrm{avg}\_\mathrm{err}}$ describes the average error for packets of a given degree, $f_{\mathrm{clean}\_\mathrm{avg}\_\mathrm{err}}$ is the average error for packets of a given degree only considering packets with an error below 100%. Together with $f_{\mathrm{non}\_\mathrm{unique}\_\mathrm{packets}}$, these metrics are evaluated for each degree, thus allowing a comparison of the degrees within a distribution function. In contrast, the overhead and the number of unrecovered packets are calculated by decoding a random subset of packets multiple times. These metrics can then additionally be used for comparison of the individual distribution function.

In this context, $f_{\delta }$ represents the impact factor for the metric *δ*, and these metrics are defined in Section 2.1. The impact factors can be used to adjust the relative importance of each metric in the overall fitness function, enabling the optimization process to prioritize certain objectives based on the requirements of the specific DNA storage setting.

The impact factors $f_{\delta }$ were determined through an iterative process of experimentation and analysis. We also determined the number of unique packets, the number of constraint-satisfying packets, and the average error to be the primary optimization targets, since these directly influence the cost of the storage process as well as its reliability. Thus, we assigned a higher factor to these metrics. However, we would like to emphasize that 1) the presented methods reliably produced improvements for all presented metrics and that 2) the choice of hyperparameters can be changed if external factors, such as the expected synthesis cost per base, will significantly change.

Since this synthetic error value uses metrics that depend on the chunk size, the file, and its entropy, its value is not normalized and can only be compared in experiments with the same parameters. A visualization as well as pseudo-code describing the optimization methods of evolutionary optimization and differential evolution can be found in the supplemental material.

By generating all packets for a series of files and chunk sizes for each distribution in each population and then calculating the error probability for each packet created, it is possible to calculate the average error probability of each degree. Then, the number of non-unique payloads is calculated. Additionally, for each experiment (per file and chunk size) multiple decoding attempts with a random subset of packets are performed, averaged, and for unsuccessful decodings with a zero packet overhead, the number of successfully recovered chunks is calculated. All these factors are used to determine both a per-degree fitness and an overall fitness of the generated distribution function.

To ensure that the optimized distribution function conforms to the requirements of being a valid and comparable distribution function for fountain codes, the following additional constraints are imposed:

1. ∀i∈[1,…,40]:deg[i]∈[0,1)
2. $\sum_{i=1}^{40}deg[i]=1$
3. deg[1]>0 (to increase decodability)
4. |[x where deg[x]>0]|>1 (more than one degree with probability >0)

Standard optimization methods like Nelder-Mead [43], gradient descent [44], or (differential) evolution [45] do not inherently satisfy these constraints, necessitating their explicit enforcement during the optimization process. To accommodate these constraints and facilitate the necessary modifications to the optimization algorithms, we implemented a custom optimization method from scratch. This provides precise control and permits further fine-tuning for specific use cases.

We enforce the outlined constraints through min-max clipping, normalization, and the introduction of random permutations for degrees with probability 0, in the case of less than 2 active degrees. Clipping is used to (a) limit the maximum error probability to 100%, (b) limit the probability for each degree to be between 0% and 100%, and (c) ensure that the probability of degree 1 is greater than 0, which increases the overall decodability. Normalization is used to ensure that all degrees sum up to 100%.

The optimization process is expected to yield the highest improvement for low-entropy input files, since combining a larger number of regions using the XOR operation leads to an increase of the entropy of the result. To develop an input-invariant distribution function, we employ seed spacing (see Section 2.3) and payload masking (see Section 2.5) using XOR operations with a pseudo-random sequence. These methods help to artificially increase the entropy of the input files, reducing the dependence of the optimized distribution function on the specific input data. Furthermore, we generate multiple distribution functions optimized for various files with distinct entropy values to cover a range of potential input scenarios.

Since we can assume that neither the encoding nor the decoding process is subject to strict time or resource constraints for DNA data storage, pre-computation of all possible packets before storing the encoded symbols in DNA can be performed, enabling the filtering of packets that violate channel restrictions and the selection of a subset of packets that minimize decoding overhead. Additionally, the anticipated high access time for DNA-based data storage relaxes the usual requirements for decoding speed, permitting the use of distribution functions that may require inactivation decoding [46], [47], [48] or Gaussian elimination [49] to decode all input symbols.

#### 2.6.1 DNA storage channel description

In contrast to data transmission over well-defined erasure channels, such as wireless broadcasts, DNA as a storage medium is subject to various predictable and unpredictable errors and mutations. Without additional error-correcting codes, DNA data storage can be best described as a bursty and input-dependent error channel. Such a channel is expected to behave differently based on the information to be transmitted, producing errors that may occur near each other for some sequences, but none for others. However, when using fountain codes with DNA, a checksum or an error-detecting and error-correcting code like Reed-Solomon can be used to simulate an erasure channel usually required for fountain codes. Formally describing the DNA storage channel model as a biological entity is challenging, hence our objective is to outline limitations that emerge when encoding data in DNA. It is important to note that due to the biological nature and diversity of DNA synthesis, storage conditions, PCR, and DNA sequencing technologies, the restrictions on stored DNA sequences can vary significantly [50].

A primary consideration is the choice of the storage medium, which can be either inside living organisms (in-vivo), such as bacteria [5], [51], [52], or outside living organisms (in-vitro) [53], [4], [21]. Since in-vivo storage is significantly more complex and less studied, we focus primarily on the optimization for in-vitro storage systems. However, all optimizations can be adapted to different constraints, such as those of a specific in-vivo experiment. The main limitations of DNA storage channels that need to be considered when designing error-correcting codes include homopolymer length, GC content, undesired subsequences, and formation of secondary structures.

The maximum homopolymer length is the maximum allowed run-length of each of the four bases and can be defined as follows:

(2) $$H(s)=\max_{i=1}^{n}\{j-i+1|j\leq n\text{ and }\forall k\in \{i\ldots j\}:s_{i}=s_{k}\}$$

This restriction can be expressed as follows: $\forall s\in S:H(s)\leq h_{max}$. Here, *S* represents the set of sequences intended for storage in DNA, and $h_{max}$ represents the maximum homopolymer length allowed. Typically, the desired maximal homopolymer length is in the range of 2 to 5 bases.

To formalize the GC content constraint, we can define:

(3) $$\forall s\in S:gc_{min}\leq \text{GC content}(s)\leq gc_{max}$$

For most DNA storage codes [16], [21], [11], the target GC content is typically between 30% to 70% and, in some cases, 45% to 55%. To ensure that the GC content does not fluctuate significantly within a sequence, several codes additionally restrict the GC content on a per-window basis.

The third limitation pertains to undesired subsequences, which are sequences that must not appear in any sequence intended for storage in DNA. The list of these sequences can vary depending on the technology used, but these sequences commonly include the primers used as well as their (reverse) complements:

(4) $$\forall s\in S,\forall k^{\prime }\in K,k^{\prime }⊈s$$

where *K* represents the set of undesired subsequences and their (reverse) complements, and *S* denotes the set of sequences to be stored in DNA.

Another frequently applied constraint involves avoiding sequences with a high likelihood of forming secondary structures. Such structures tend to be disadvantageous for various processes, including DNA sequencing and random access through PCR [11], [54]. A common approach to estimate the probability of secondary structure formation involves the calculation of the free energy of the sequence, measured in kJ mol^−1^. However, the complex nature of this calculation could significantly slow down the iterative optimization process. Given that adhering to the previously mentioned restrictions is adequate for reliable data storage in and retrieval from DNA [55], [56], [15], [21], we decided to omit this particular restriction. Nevertheless, it is an option to include this restriction if needed.

Throughout the DNA data storage process, random errors such as insertions, deletions, substitutions, and the removal of complete sequences can occur. While these errors can be mitigated by employing robust inner codings, such as Reed-Solomon [20] or arithmetic coding [21], the erasure of complete sequences can be addressed through fountain codes by adjusting the overhead to compensate for the anticipated erasure rate.

Due to the complex biological nature of DNA storage systems, it is difficult to develop a simple mathematical model that accurately describes the DNA storage channel, such as an additive Gaussian (white) noise channel or a linear filter channel. As a result, a direct algebraic approach to optimizing the distribution functions of fountain codes for DNA storage systems is unlikely to be feasible.

#### 2.6.2 Selecting a distribution function

The choice of a distribution function is a critical factor in optimizing fountain codes for DNA data storage. Using multiple input-optimized distribution functions offers advantages, but it raises the question of how to make the chosen function available to the decoder. Storing the distribution function directly in DNA could ensure successful decoding. However, this approach has significant drawbacks in terms of storage density and error probability. The commonly used distribution functions are defined by a list of 40 floating-point numbers, each using 8 bytes. Storing this directly in DNA would require 1,280 additional nucleotides, or about 5 sequences of 256 nt each (not including indexing), given the typical sequence length limit of approximately 300 nt. Compression could reduce the size of this data, but it would also make the system highly vulnerable to mutations - a single unhandled error could prevent recovery of the entire distribution function, rendering the stored data inaccessible. Furthermore, translating the compressed representation to DNA's quaternary code could produce sequences that violate biochemical constraints, necessitating additional error-correction coding. Since a loss of the distribution function would result in a total loss of the information stored, an overhead of 10 additional sequences with about 300 nt each would be more realistic. Furthermore, the relative quantity of these sequences should be reasonably higher than for the payload to guarantee that all of them can be sequenced and clustered without errors. Besides the challenge of storing the distribution function into DNA, a further challenge would be to distinguish the actual payload from the stored distribution function. For this challenge, one could either reserve a seed (e.g., seed 0) which then indicates the stored distribution function, or use a different primer to establish a clear separation of payload and distribution function. However, if future research regarding the optimization of fountain codes for DNA storage uses more than 40 degrees or a higher resolution, these problems would increase even further.

Thus, this approach would have the drawback of reducing the storage density, increasing the error probability, and potentially requiring additional coding if the selected and serialized distribution function violates any critical rules. Considering the limited factors influencing the optimization of fountain codes and the potential high impact of errors in the sequences that store the distribution function, we propose the following zero- or low-overhead methods to indicate the selected distribution function:

##### 2.6.2.1 Using a predefined set of distribution functions

Manually tailoring a distribution function for each file to encode might yield slightly improved results with respect to the previously defined metrics, but this method has several drawbacks. It not only requires manual optimization for each stored file, but also introduces the disadvantages stated above.

A better approach is using a predefined set of distribution functions. With a limited number of possible distribution functions, the transmission of the used distribution function can be accomplished by additionally storing the ID of the distribution function. However, this method reduces the storage density, since this information must be stored in each DNA strand. Alternatively, a single short DNA sequence could be synthesized to indicate which distribution function was used. As long as the length of this sequence significantly differs from the sequences containing the payload, prior analysis to decode the used distribution function would be possible. Nevertheless, this method has several challenges:

1. A single DNA sequence that encodes the used distribution function introduces a single point of failure if a mutation or an erasure of the sequence occurs.
2. Even in the presence of multiple copies generated by PCR, it must be ensured that the original sequence was synthesized correctly.
3. The complexity of the sequencing pipeline increases, making the initial selection by sequence length more challenging.
4. The storage density is slightly reduced, impacting cost-effectiveness.

Depending on the number of distribution functions and the presence of a file-wide checksum in a header, it might be reasonable to decode the stored data with all possible distribution functions and determine the correct one using the checksum. Although this increases the resources required for decoding, the additional runtime cost is likely to be lower than the cost of storing additional bases in each DNA sequence. The drawback of this method lies in the inability to determine the correct distribution function in the event of an error, resulting in an incorrect checksum for all versions.

##### 2.6.2.2 Mapping DNA subsequences to predefined distribution functions

**Fig. 4:**
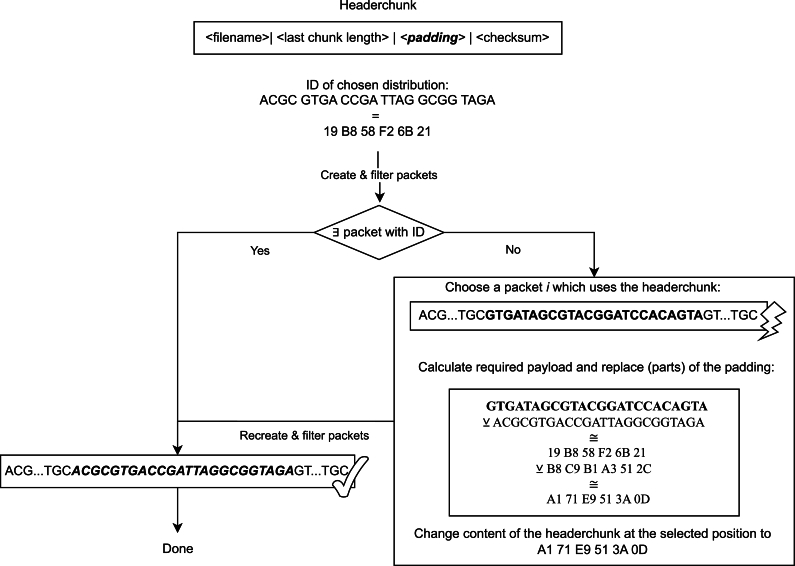
Process of ensuring the existence of a DNA sequence with a given codeword. Only packets using the header chunk will need to be recreated.

Since the use of an optimized distribution function increases the number of packets that conform to defined restrictions, it becomes feasible to store the ID of the used distribution function using specialized rules. This requires defining a set of DNA subsequences, each mapping to a predefined distribution function. During packet creation, all codewords except the one mapping to the used distribution function are disallowed. This method allows us to utilize the presence and absence of specific subsequences to directly determine the used distribution function. However, the length of these identifying DNA subsequences is important. If the sequences are too short, there is an increased probability that these sequences randomly arise in the encoded packets, which could lead to a substantial drop of otherwise valid sequences. In contrast, using excessively long identifying subsequences may make it impossible to generate any packets containing the desired subsequence. In this case, it is possible to utilize the unused space in the header or the padding of the last chunk to embed binary data. Once encoded using the distribution function, this data would produce the desired subsequence. An example is shown in Fig. 4.

If this method is not feasible due to insufficient space in these regions, introducing an additional chunk is an alternative. This chunk could include the desired subsequence or a matching derivative, provided that this chunk is not included in a degree 1 packet. However, the inclusion of an extra chunk for this purpose would decrease the overall information storage density of the DNA data storage system. Therefore, it is worthwhile to explore if selecting a slightly less optimal but still effective distribution function could provide better performance. Importantly, this approach of encoding predefined identifying subsequences into the data-containing DNA strands is not limited to specifying the distribution function. It can be extended to efficiently store any type of metadata relevant to the encoded data, without sacrificing information density. This can be achieved by defining a mapping function *f* that translates an enumerable set of metadata tags into unique DNA subsequences, which are then deliberately included or forbidden in the synthesized DNA strands, as shown in Fig. 4.

### 2.7 Applicability of the proposed methods

The proposed optimization methods are specifically designed to address the distinct error characteristics and constraints of the DNA storage channel, which differ from a standard erasure channel model. Seed spacing (Section 2.3) helps to decouple the structure of the seed from the likelihood of rule violations, reducing the influence of seeds that would introduce errors. Payload masking (Section 2.5) introduces uniformly distributed random sequences into the payload, increasing entropy and reducing the probability of rule-violating sequences. Optimizing the fountain code distribution function (Section 2.6) creates degree distributions tailored to the DNA storage channel, balancing encoding efficiency, error correction, and storage density. Together, these methods account for the complex, bursty, and input-dependent nature of errors in DNA storage, going beyond the assumptions of a simple erasure channel to enable more reliable and efficient DNA data storage. The differing error characteristics of the DNA storage channel are handled by using per-packet error detection and correction schemes, reducing the error probability through randomizing the payload and spacing out the seed as well as creating more unique and less error-prone packets through an optimized distribution function. Other factors such as the inability to send more packets due to the storage architecture compared to a transmission-based channel are handled through the use of redundancy, which is optimized by generating more unique packets, requiring less overhead and being able to retrieve a larger percentage of chunks in the case of a failure to decode. This is achieved through the optimization of the distribution function.

## 3 Results

In this section, we evaluate the impact of each proposed method and assess its overall benefits. Due to its inherent limitations, the method of increasing entropy by compressing or encrypting the input (see Section 2.4) is not evaluated individually.

### 3.1 Seed masking

The method of seed masking is briefly compared to the approach of Erlich and Zielinski [15]. Their implementation uses a linear feedback shift register to pseudo-randomly generate unique seeds in the range from 0 to $2^{32}-1$. This approach has an equal probability of returning a seed that does not violate the restrictions of the channel, but it has two major implications: (1) since the chosen polynomial operates on 32-bit numbers, it is not possible to use shorter or longer seeds; (2) this approach complicates the possibility of parallelizing the encoding process, since assigning a range of seeds to use to each worker would require pre-computing the initial position of the linear feedback shift register.

If all possible packets in the 4-byte seed range have to be generated, the use of seed masking, the approach used in DNA Fountain [15], and the sequential processing of all possible seeds, have nearly the same performance. In this case, only minimal deviations regarding the number of computations performed in each step can be expected. Taking the use of sequential seeds as a baseline, the seed masking approach introduces a single additional XOR operation per seed, whereas the approach by Erlich and Zielinski requires a full iteration of the linear feedback shift register. Furthermore, we generate seeds using a Mersenne Twister [57] implementation, which supports versions with a period length of up to $2^{19937}-1$, allowing its use for files that require more than $2^{32}-1$ packets.

In a nutshell, seed masking is suitable to reduce the burstiness of rule-violating packets, as outlined in objective **MinBurstError**, but does not improve the overall performance regarding error probability or expected overhead.

### 3.2 Seed spacing

To evaluate the method of seed spacing (Section 2.3), we generated all possible packets for the English text of the *Sleeping Beauty* novel [58] using a 2-byte unsigned seed space given the default distribution function and a chunk size of 40 bytes (resulting in a total of 122 chunks) while varying the interleaving values. The *Sleeping Beauty* novel has a total size of 6,641 bytes with an entropy of 4.36472 (1.94101 when using the entropy calculation for the four DNA bases). Since this file has a moderate entropy, it can be expected that packets of a lower degree are more likely to not pass the restrictions. Further details about the files used during the experiments performed can be found in the Supplement. In the following experiments regarding seed spacing, we applied the payload masking approach for all experiments, including the baseline. This minimizes the effect of the used file, and the effect of seed spacing can be observed without any noticeable impact from the used file. Furthermore, since for each experiment the generated payload for each seed is the same, we ensure that only seed masking influences the result.

**Fig. 5:**
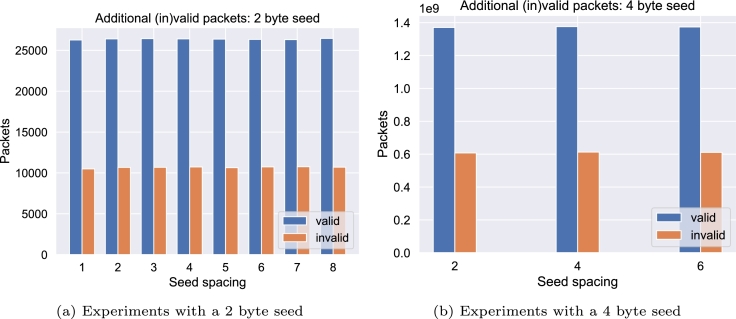
Impact of seed length and seed spacing for various intervals. Each experiment depicts the number of additional valid and invalid packets as a direct consequence of seed spacing compared to the baseline using no seed spacing. Both experiments assume a maximum homopoylmer run of 3 as well as a GC content between 40% and 60%.

Increasing the seed spacing further does not significantly improve the number of valid packets, since homopolymers resulting from the seed are efficiently broken up even by minimal spacing. Given that the number of initially rule-abiding packets were 14,133 (21.6% of all possible packets) for the 2-byte experiment, as shown in Fig. 5a, and 2,509,354,661 (58% of all possible packets) for the 4 byte version, as shown in Fig. 5b, this approach alone was able to increase the number of possible packets by 111.57% (+15,769) and 30.42% (+763,349,290) respectively, which can be directly translated into increasing the maximum file size to store in a single encoding. Using seed spacing, 45.6% (76.2% for the 4 byte version) of all possible packets complied to the restrictions. Restricting the maximum allowed homopolymer run to 2, only 28,598,530 (0.67% of the total) packets passed the restrictions without any seed spacing. In this case, seed spacing was able to increase the number of valid packets to 1,336,003,970 (using a seed spacing of 4) which is equivalent to 31.1% of all possible packets. A visualization can be found in the supplemental material. Since all experiments using the same seed spacing produced the same set of packets, it can further be seen that, for example, for the 4-byte experiment using a seed spacing of 4 (Fig. 5b and the corresponding figure in the supplement), out of the 3,272,703,951 packets that fulfilled the restriction having a homopolymer run of 3 or less, 1,336,003,970 also had a homopolymer run of 2 or less. Besides the influence of the homopolymer, additional restrictions such as a windowed GC content or undesired motifs are expected to be improved due to the use of seed spacing. However, since they result in a significantly smaller number of rule violations, they were not further analyzed in this experiment.

It is important to note that seed spacing should not exceed a certain value, since this could lead to a scenario in which most of the seed is positioned at the end of the sequence. This can occur if the spacing of the seed exceeds $\frac{\left|\text{payload}\right|}{\left|\text{seed}\right|}+1$, where |seed| represents the length of the seed in bases. In this case, more than one base of the seed will be positioned at the end of the sequence without any spacing.

### 3.3 Payload masking

The effectiveness of payload masking depends on the payload and its entropy, but the use of payload masking as described in Section 2.5 resulted in an improvement for all files tested.

**Fig. 6:**
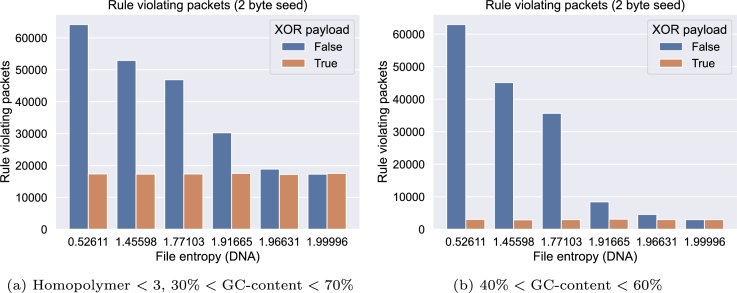
Rule violating packets for files with varying entropy. XOR'ing the payload with i.i.d. random data leads to a reduction in the number of rule-violating packets. In each experiment, 2^16^ = 65,536 packets were generated.

As shown in Fig. 6a, the combination of the payload of each packet with a pseudo-random sequence effectively reduces the number of packets that violate channel restrictions. In this experiment, the maximum homopolymer length was set to 4, the overall GC content and the 50-base windowed GC content were restricted to be between 30% and 70%. Furthermore, the presence of 10 different Lox sites and two Twister adapters, typically required for various biological processes and preparation steps for next-generation sequencing, was prohibited. The list of restricted subsequences can be found in the supplement.

Fig. 6b shows the results of a similar experiment, but with a GC content (both global and per window) between 40% and 60%. It is evident that the entropy of the file to be encoded significantly influences the potential improvement achievable by this method. Low-entropy data, such as black and white and grayscale bitmap images (entropy: 0.52611,1.45598, and 1.77103) yield a substantial number of rule-violating packets without using payload masking, and the improvement is more pronounced. On the contrary, the improvement for raw text (1.91665) and a JPEG encoded image (1.96631) is less noticeable. No statistically significant improvement was observed for a compressed ZIP file containing a raw text file and a PDF file (1.99996). Although the size of the used files vary, by choosing a fixed chunk size of 40 bytes for all files, together with the fixed distribution function, this did not have an impact on the experiments. Additional information about all files used can be found in the supplement.

This result suggests an alternative to XOR'ing the payload, which involves compressing the input using a suitable compression algorithm. Although this method can yield similar results with a smaller total size, this method is not generally feasible. As described in Section 2.4, the use of compression methods not only restricts users to specific use cases, but may also reduce the data recovery rate in the case of an unsuccessful decoding [59]. Moreover, to achieve a storage method comparable to existing technologies, it is crucial to provide the same freedom of storage that conventional methods offer. Therefore, preference should be given to utilizing the XOR operation on the payload over file-wide compression methods.

### 3.4 Optimizing the distribution function

In the following, we present the results of optimizing the distribution function, showing how the optimization methods improve the various metrics over the course of 500 generations.

Since DNA data storage does not have the same requirements as live data transmission, we employed a Gaussian elimination decoder, which, if possible, guarantees a successful decoding at the cost of higher complexity. However, hybrid decoding approaches, such as an inactivation decoder [48] that combines the benefits of a belief propagation approach with Gaussian elimination, can be used to reduce the complexity of the decoding process. The time complexity is cubic in the number of inactivations [48].

Our optimizations are based on minimizing the synthetic error value calculated for each distribution function, as described in Section 2.6 and Equation (1). In each iteration, a population size of 100 distributions was used and 10 randomized decodings were performed for each encoding to calculate the average overhead required to decode the encoded data (**MinAvg**) and the average number of unrecovered packets (**MaxDecode**). To avoid overfitting based on the input file and chunk size, all combinations of selected input files and chunk sizes were generated for each distribution function. The synthetic error value was therefore calculated on the set of input files and chunk sizes for each distribution function in the population. Initial tests indicated that using a high-entropy input file avoids common overfitting pitfalls. Therefore, we used three different values for the chunk sizes: 40, 60, and 80 bytes, corresponding to DNA sequences of length 160, 240, and 320 nucleotides, using a single AES-encrypted file of high entropy. In a first evaluation, we encoded the AES-encrypted version of the *Sleeping Beauty* novel with a 4-base seed spacing and the payload masking method. This allows us to provide an estimate of the potential improvements for any high-entropy input. Since any file can be compressed or encrypted, the result of this optimization may be applied to any input file. Additional optimization results for other files and entropy groups can be found in the supplement.

**Fig. 7:**
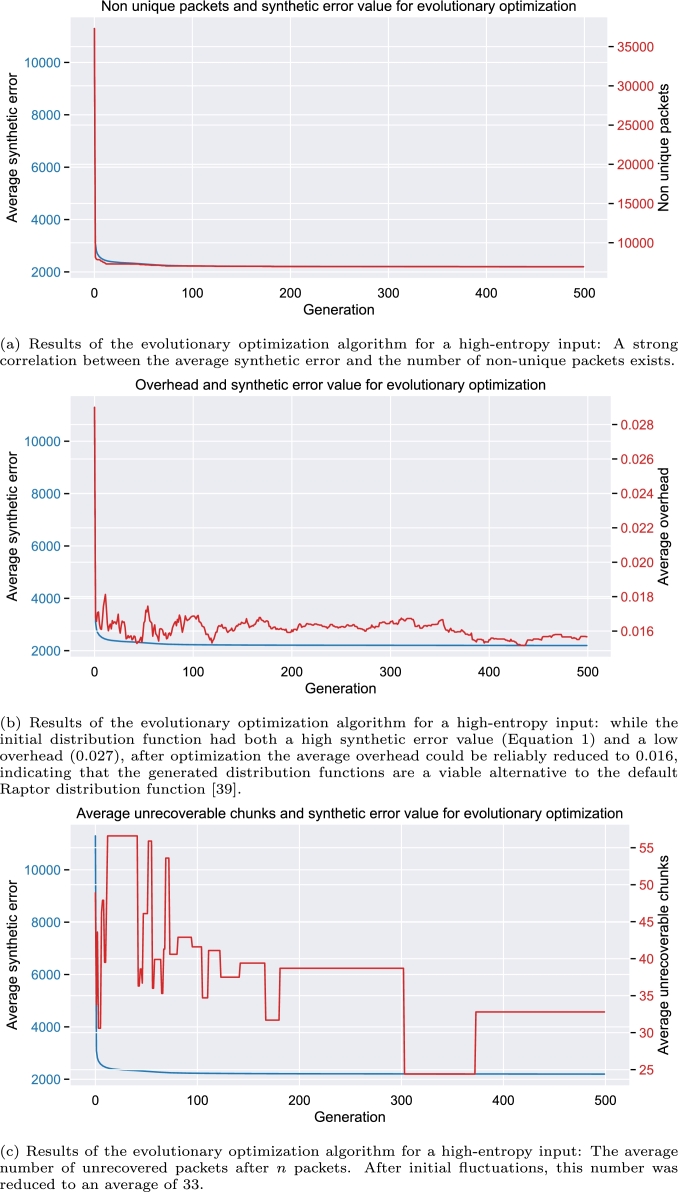
Results of evolutionary optimization for each generation.

As shown in Fig. 7a, a strong correlation is present between the number of non-unique packets and the synthesized error value. This correlation is expected due to the high-entropy input and the high impact factor of 0.3 towards the synthetic error value.

Fig. 7b shows a decrease in the average overhead required. Even when using a factor of 0.4, this is less noticeable in the final synthetic error value, yet the optimization algorithm starts to focus on optimizing this objective. This behavior becomes particularly evident after the first 100 generations as well as after approximately 350 generations, where most other optimization goals already show no further improvements.

For the number of rule-conforming sequences, the initial number of about 10,000 sequence is quickly increased to slightly below 26,000. After this initial improvement, only a modest but consistent improvement can be observed. A visualization of the improvement over the generations can be found in the supplemental material. Since the probability of rule violations is correlated with the entropy of the produced packets, which, in turn, relates to the entropy of the input and the degree used during creation, substantial improvements were not expected.

As described in Section 2.1, the average number of unrecovered chunks after *n* received packets (equivalent to objective **MaxDecode**) should be minimal. This value depends on the set of selected packets {p⊂P||p|=n}, and therefore, for each distribution, an average of 10 runs (configurable for each experiment) per distribution was generated in each generation. With a factor of $f_{\mathrm{unrecovered}\_\mathrm{packets}}=0.1$ towards the synthetic error value, as described in Equation (1), we primarily used it as a preventive measure against degenerated distributions.

Fig. 7c depicts the average count of unrecovered data chunks after decoding *n* randomly selected packets, specifically focusing on the distribution with the lowest synthetic error value in each generation. Following initial fluctuations, the optimization process effectively prevented an increase in unrecovered data chunks in instances of an unsuccessful decoding, and even resulted in a slight reduction.

Both evolutionary optimization and differential evolution show improvements compared to the initial distribution. However, differential evolution quickly converges to a local minimum, resulting in minimal changes throughout subsequent generations. In contrast, evolutionary optimization shows steady improvements over all 250 generations. This behavior may be attributed to hyperparameter choices in differential evolution, which are explored in the supplementary material. Additional figures for experiments conducted with lower entropy input files, including the generated distribution function as well as a graph comparing the overall optimization for both evolutionary optimization and differential evolution, can be found in the supplemental material.

**Fig. 8:**
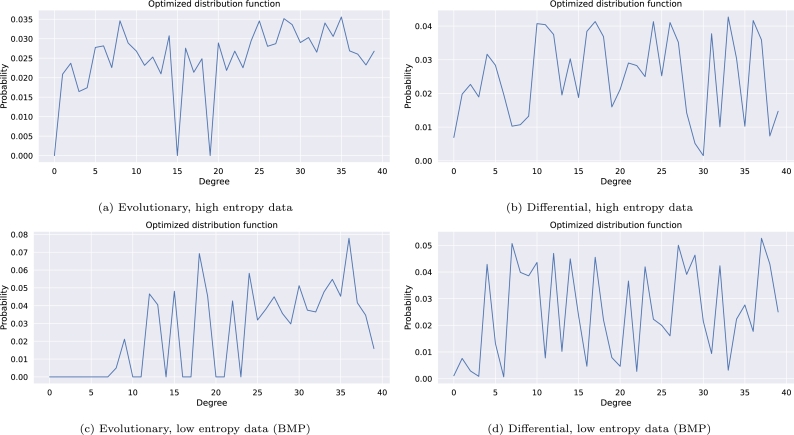
Optimized distribution functions for the AES-encrypted and masked input, as well as for a low entropy input file. For low entropy input, higher degrees were preferred.

The optimized distribution functions are shown in Fig. 8. In the high-entropy experiment, the results revealed a relatively uniform distribution. Although evolutionary optimization (Fig. 8a) showed only a minor increase in the probability of higher degrees, differential evolution (Fig. 8b) did not exhibit this behavior. In contrast, in experiments involving low-entropy input, the resulting distribution functions displayed reduced the utilization of the low degrees. In this case, the evolutionary optimization algorithm (Fig. 8c) reduced these degrees significantly while maintaining or improving the defined optimization objectives, compared to differential evolution (Fig. 8d). However, compared to the initial distribution function used for the Raptor code, a substantial shift towards a more uniform usage of all degrees can be observed.

We notice an increase in average overhead as the average error rate decreases, but it is crucial to acknowledge that this observation predominantly applies to live data transmission scenarios. In contrast, when working with offline storage media, such as DNA storage, it becomes possible to strategically select specific combinations of data packets to optimize a successful decoding while minimizing the required overhead. This strategy hinges on the initial packet selection meeting two critical conditions: (1) strict adherence to all imposed restrictions, and (2) maintaining low overhead across all possible combinations. The unordered (estimated) overhead during subsequent optimization can be safely disregarded. It is essential to emphasize that, while determining the optimal packet combination for minimal overhead poses computational and mathematical challenges, it remains both feasible and beneficial. Furthermore, when considering the economic aspects of DNA storage, this optimization process becomes even more compelling. This approach offers increased robustness against an unsuccessful decoding and enhances the (partial) recoverability in scenarios where full decoding is not achieved.

**Fig. 9:**
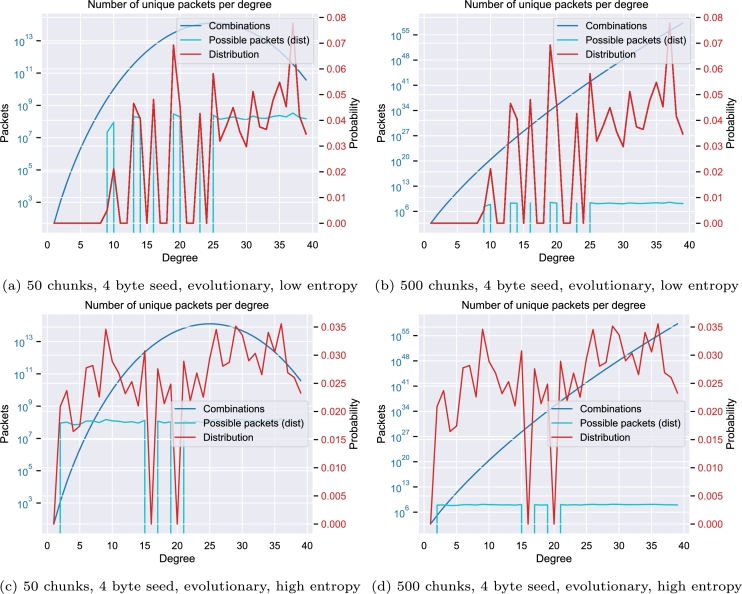
The maximum number of unique packets, shown for the optimized distribution functions of Figs. 8a and 8c.

As shown in Fig. 9, all optimized distribution functions exhibit a significantly improved distribution of possible packets regarding the total number of available combinations. Given these improvements, the probability of generating duplicate packets is reduced compared to the Raptor distribution and the robust soliton distribution used in DNA Fountain, which are depicted in Fig. 3 and in the supplemental material, respectively.

To summarize, optimizing the distribution function leads to several improvements, including a reduction of non-unique packets, a reduction of the average overhead required to decode a file, a significant increase in the number of rule-abiding packets, and a reduction of the number of unrecovered packets in the case of an unsuccessful decoding. The increase in unique packets is important with respect to the cost of synthesizing sequences. The reduction of the average overhead required for decoding also improves the information rate, which reduces the storage cost or alternatively increases the reliability against errors. Additionally, since our method is based on a rule system that assigns floating-point values for the error probability, the reduction of the average error, which is a result of increasing the number of rule-abiding packets, will most likely result in less errors. Although a reduction of the number of unrecovered packets in the case of an unsuccessful decoding does not improve the performance of the coding schemes in the regular case, it is critical in the case of a partial failure to decode.

### 3.5 Comparison with existing codes

The previous results demonstrate the improvements of our optimization methods regarding the chosen metrics compared to the baseline. To further evaluate our findings, we conducted a series of experiments comparing our optimizations with existing codes, namely DNA Fountain [15], NOREC4DNA [16], and the code proposed by Grass et al. [4], using a subset of files of the NapierOne dataset [60]. The selected subset contains several real-world files in the following formats: *BMP*, *TXT*, *ZIP*, *XLSX*. Details about the used files can be found in the supplement. For the fountain code schemes, each file was encoded with a GC content limit between 40% and 60% as well as a maximum homopolymer length of 3. The number of packets to generate for each file was set to three times the number of chunks and each packet had a length of 117 nt.

#### 3.5.1 GC content

Investigating the generated packets for the files of the NapierOne dataset regarding the GC content, all fountain code methods outperformed the code proposed by Grass et al., which does not impose a strict GC content. Furthermore, all Raptor-based methods, including the baseline implementation from NOREC4DNA [16], outperformed the DNA Fountain approach in all experiments. A detailed comparison of the various codes and distributions regarding GC content of the generated sequences for different file types can be found in the supplemental material. For all schemes based on fountain codes, it would be possible to further limit the GC content and thus produce perfectly aligned packets at the expense of an increase of rule-violating packets. Likewise, the GC content for the output of the code presented by Grass et al. cannot be controlled; the limited number of generated sequences and the inability of using parts of the produced output limit the universal applicability of this approach.

Taking all encoded packets into account, as shown in Table 1, the distribution function optimized for low entropy files performs the best regarding the mean value. However, for the standard deviation, the distribution function optimized for high-entropy input slightly outperforms this distribution. Both optimized versions outperform the baseline code and the two other encoding schemes.

**Table 1:** Computed GC content over all generated packets for the used files in the NapierOne dataset.

Method	Mean	Std
Low entropy - evo	0.4999932	0.03357297
High entropy - evo	0.5000155	0.03356818
Raptor (baseline)	0.4993714	0.03539878
DNA Fountain	0.4986722	0.04281341
Grass	0.5037254	0.04100128

Using the optimized distribution functions, the mean GC content is more balanced and the standard deviations are significantly smaller than those of the baseline and the codes used for comparison. This will effectively result in a larger number of usable encoded sequences, which in turn results in a larger maximum file size for any given seed range. Additionally, this will also reduce the error probability for the actual DNA data storage process, which might arise from unfavorable GC contents. This behavior can be best explained by the increased average degree for the generated packets, which can increase the average entropy.

#### 3.5.2 Secondary structure prediction

Although we did not perform optimization with the goal of avoiding secondary structures by evaluating the minimum free energy (MFE), we evaluated the created distribution functions for this metric and compared them with the sequences generated by the code of Grass et al. and DNA Fountain. All evaluations were performed using *ViennaRNA* [61] with the default parameters. Again, since the code of Grass et al. produces sequences of 117 nt in length, the typical limit of 300 nt was not used, but instead DNA Fountain and our method were limited to this length.

**Fig. 10:**
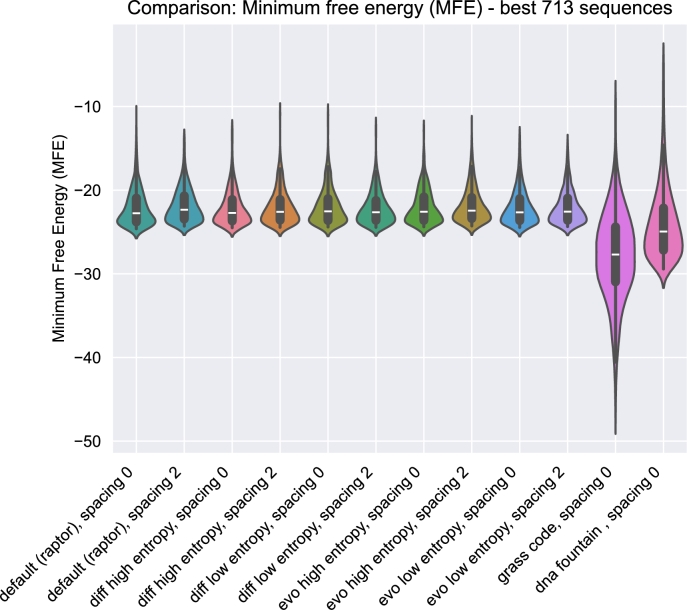
Minimum free energy for the *Sleeping Beauty* file for the various distribution functions without using payload masking. All experiments contain 713 sequences.

Since the code proposed by Grass et al. produced 713 sequences, we selected the best 713 out of 5,000 randomly generated sequences for the fountain code schemes in Fig. 10. Due to the increased code rate, our method as well as the DNA Fountain code could successfully decode from approximately 289−400 sequences (both codes split the file into 289 chunks), thus allowing an additional refinement of the selection of sequences. All of our implemented solutions outperform the DNA Fountain implementation, as well as the code proposed by Grass et al. The 2-base seed spacing outperforms the default implementation. Furthermore, the distribution optimized for low entropy input using evolutionary optimization and a 2-base seed spacing (mean MFE: −21.47) slightly outperforms the other custom distribution functions, highlighting the successful optimization for such files. While secondary structure prediction is a complex task and the minimum free energy estimation was not part of the fitness function, the results show that both the use of seed spacing and distribution optimization were capable of producing measurable improvements for the avoidance of secondary structure formations. In addition, the optimized functions are more stable, indicated by less variance in both the positive and the negative direction. Otherwise, no significant improvements for the custom distribution functions can be observed.

To evaluate the effect of seed spacing and increasing the entropy of the encoded packets, we compared the DNA Fountain implementation presented by Erlich and Zielinski [15] with a version modified by us which includes these methods. In this experiment, we generated sequences with a length of 300 nt. For this experiment, the mean minimum free energy for the original implementation is −90.22, whereas the version with seed spacing and payload masking has an improved mean value of −83.41. Furthermore, the modified version resulted in a more narrow distribution of the MFE values than the original implementation. While only a 5,000 sequences long subset was generated and analyzed, this set was randomly selected throughout the seed space using the Mersenne Twister implementation used in DNA Fountain, thus ensuring a random selection of seeds over the complete range. For both experiments, the same set of rules was applied, resulting in the two rule-abiding sets of 5,000 sequences. A detailed violin plot of these experiments can be found in the supplemental material. The improved MFE for the modified version of DNA Fountain including our seed spacing and payload masking methods further highlight that they effectively improve the quality of the generated packets for any DNA coding scheme based on fountain codes.

#### 3.5.3 Unique packets

In the following, we analyze the behavior of the optimized distribution function against the **MaxUniquePackets** goal. To evaluate the effect of the presented methods in terms of their ability to produce unique sequences, we encoded two files with vastly differing entropy values using all comparable methods. For this purpose, all 2^16^ packets were generated without filtering out sequences that violated any rules. These sequences were then clustered using “UCLUST” [62] to get the number of singletons in each set. The identity value was chosen such that sequences that only differed in the seed were treated as equal. Additionally, DNA Fountain was modified to allow the generation of 2 byte seed values. The code proposed by Grass et al. cannot create additional packets and is therefore not considered here. Since the two files have different sizes and thus a different number of chunks, the absolute numbers cannot be compared with each other. The number of unique clusters is an indicator to determine duplicates that only differ in the seed. Thus, it can help us to detect sequences that do not increase the error resilience and therefore only lower the code rate, which directly increases the cost of the storage process. Besides the potentially increased cost and missing contribution towards decoding, reducing the number of packets with the same payload increases the total number of available sequences for decoding.

As argued in Section 2.6, it can be expected that distribution functions with a higher average degree are less likely to produce duplicate packets. Table 2 indicates that the distribution function optimized for low entropy input can significantly increase the number of unique sequences from an initial 57,992 up to 64,025. With this 10.4% improvement, the code produced unique packets for 97.69% of all packets in the seed range. This increase in unique packets can be directly translated into a 10% increase of the maximum file size that can be stored without the need for reducing the code rate by increasing the seed size. Furthermore, the use of distribution functions optimized for high entropy files improved this metric, although it was not as significant as the evolutionary or differential evolution-based optimization for the low entropy files. When using the compressed file “Dorn.zip” as a high entropy input, the impact of using the optimized distribution function was less significant. However, all optimized distribution functions outperformed the default Raptor distribution as well as the default configuration of DNA Fountain. Since both the low entropy BMP file and the compressed and thus high entropy ZIP file achieved the best results with the respective distribution functions, the optimization successfully improved the distribution functions for the respective entropy ranges. For this experiment, the optimized version was able to generate unique packets containing new information for 99.99% of all produced sequences. Since payload masking was used for the experiments using the NapierOne dataset, the generated output sequences were all unique. However, since the underlying packets used may not be unique, the use of payload masking does only increase the number of unique clusters, yet it does not prevent the problem of encoding multiple packets containing the same information. The results presented in Table 2 thus apply even when the payload is masked. As a result of this increase in the number of unique packets, larger files can be stored in DNA using a smaller seed size, which in turn can increase the code rate and thus significantly reduces the cost. Additionally, when not using the seed masking approach, this method can decrease the number of sequences with a small distance from each other, which might lead to other problems, such as cross-hybridization or unspecific bindings of important binding sites [63].

**Table 2:** Comparison of the number of unique sequences / clusters for each configuration. During each experiment, a total of 65536 sequences were generated. Each file was split into chunks of 40 bytes length.

File	Distribution function	Unique clusters
logo_mosla_bw.bmp	Raptor (default)	57992
	Evolutionary, low entropy	64025
	Evolutionary, high entropy	62068
	Differential, low entropy	63152
	Differential, high entropy	61100
	DNA Fountain	41369

Dorn.zip	Raptor (default)	63536
	Evolutionary, low entropy	65451
	Evolutionary, high entropy	65481
	Differential, low entropy	65461
	Differential, high entropy	65466
	DNA Fountain	63946

#### 3.5.4 Overhead

To analyze the expected overhead required for a successful decoding, we encoded each file using DNA Fountain [15], the Raptor code implemented in NOREC4DNA [16], and our optimized versions using seed spacing, payload masking, and the custom distribution functions presented in Figs. 8a and 8c. For each encoded packet, the decoding was performed 5 times, where each repeat used a different randomly selected list of packets. Furthermore, for our implementation we repeated this for 2,3, and 4 RS symbols per strand (decreasing the chunk size to obtain sequences of the same length over all experiments). As a result, 15 repeats were performed per file for the Raptor-based implementations. The average required overhead is an important metric for fountain code schemes, highlighting the resilience of such a code against random dropouts and errors.

As shown in Table 3, the mean overhead of all files for the DNA Fountain method is significantly higher than for the Raptor-based methods, highlighting the significant improvements Raptor-based coding schemes can achieve. The large spread for the DNA Fountain approach can be explained by the differing number of chunks used to encode the various files. For example, the DNA Fountain approach required an overhead of 4,198 packets to decode a file encoded into 50,155 chunks. For the same file, except for one run with an overhead of 6 packets, all runs using both the optimized distribution functions for low entropy and high entropy required no additional overhead to decode. Since each required overhead reduces the maximum file size, the code rate, the error correction capability, and the efficiency of the en- and decoding process, the Raptor-based coding scheme alone represents a significant improvement compared to the LT-based DNA Fountain approach.

**Table 3:** Mean overhead of the encoded files for the different methods.

Method	Mean overhead	Std	Max
DNA Fountain	1032.433333	1434.449181	4198
Raptor (baseline)	7.100592	9.840178	40
Low Entropy - evo	3.295597	4.4102202	20
High Entropy - evo	2.503106	2.817632	13

The results for the optimized distribution functions shown in Table 3 indicate that the distribution function optimized for low entropy input files produces results with a slightly higher average overhead. This behavior can be explained by our fitness function to minimize the average error and maximize the number of unique and constraint-adhering packets (see Section 2.6). Since the low entropy input restricts the optimization and the possible search space significantly stronger than input files with a higher entropy, this behavior was expected. However, by halving the mean overhead, standard deviation, and the maximum overhead observed in our experiments compared to the baseline implementation even for the less optimal version, it is evident that the proposed optimization can improve the distribution function towards the defined goals.

As an additional metric obtained during testing distribution functions regarding overhead, we evaluated the average percentage of unrecovered chunks when decoding without any overhead, which is equivalent to the **MaxDecode** objective. When comparing the percentage of unrecovered chunks at an overhead of 0, both optimized distribution functions outperformed the default distribution. Here, the high entropy distribution achieved a mean of 47.24% and the low entropy version a mean of 53.59%. Thus, both optimized distributions outperformed the Raptor distribution that exposed a mean percentage of 63.45%. The better performance of the high entropy distribution can be explained by the fact that the low entropy distribution also had to focus on optimizing against low entropy input, which resulted in the absence of lower degrees in the distribution. While this metric does not have a direct impact in the case of a successful decoding, maximizing the value as shown for the two evaluated distribution functions can significantly increase the amount of information that can be retrieved in the event of a partial data loss or an error rate higher than anticipated. By combining this improvement with the increased number of unique packets, the improvements presented in this area could further be used to implement unequal error protection by selecting a subset of encoded sequences that ensure that certain parts of an encoded message can be decoded with a high probability. A box plot comparing the result of evolutionary optimization for high entropy and low entropy files with the baseline distribution can be found in the supplemental material.

### 3.6 Summary

The presented methods consistently demonstrate lower average error rates across all tested files. The main impact in this area can be attributed to our novel methods of seed spacing and payload masking, which effectively mitigate the impact of the used seed and entropy of the generated packets. The lower overhead required by our code, especially for larger files, leads to a more efficient coding process. This efficiency can be attributed to the tailored distribution function that better balances the trade-off between low-degree and high-degree packets. This reduced overhead translates directly to increased storage density, a higher code rate, and thus reduced costs for the DNA data storage process. The superior performance of our code for partial recovery scenarios is particularly noteworthy. The ability to recover a higher percentage of the stored information when full decoding is not possible is crucial for DNA data storage applications, where data degradation over time is a concern. This improvement is also a result of the optimization of the distribution function towards this metric. While the presented methods for the optimization of the distribution function with the chosen impact factors for the selected metrics may lead to a slightly increased complexity during decoding, the non-real-time nature of DNA data storage makes this trade-off worth considering. This increased complexity is mainly due to the higher average degree for each generated packet and due to the linear en- and decoding complexity of the underlying Raptor codes. The increased complexity is expected to be relative to the increase of the average degree. Additionally, by changing the impact factors, the complexity may further be reduced.

During our evaluation, we compared our results with the comparable and well established coding schemes DNA Fountain and the code developed by Grass et al. Here, it is important to note that in theory the use of seed spacing could be applied to almost any existing coding scheme, but payload masking and especially distribution function optimization will be only beneficial for coding schemes incorporating fountain codes. An example for such a coding scheme is the DNA-Aeon code [21] that uses our NOREC4DNA [16] scheme as an outer code. Thus, the presented improvements directly translate to DNA-Aeon as well.

## 4 Discussion

We presented several methods for optimizing fountain codes to improve their overall usability in DNA data storage systems. Apart from generally applicable customizations of fountain codes, such as (a) seed masking, (b) seed spacing, (c) increasing the input entropy by compression or encryption, and (d) payload masking, we (e) particularly proposed optimization algorithms to create tailored distribution functions for DNA data storage, which is novel in this context. These methods were accompanied by a novel approach for storing small metadata by embedding or restricting certain subsequences in the generated packets, allowing a decoding without prior knowledge about which distribution function was used. We described optimization objectives specific to DNA data storage using fountain codes and explained the main differences between the use of fountain codes for reliable data transmission in communication systems and in DNA data storage. We evaluated the proposed methods for optimizing fountain codes for DNA storage in terms of several metrics relevant for the DNA storage channel to show their effectiveness. In particular, we examined the impact of an added seed on the adherence to the rules for each generated DNA sequence. Furthermore, we explored the influence of the input entropy on the defined metrics and elaborated techniques to address varying or low-entropy inputs.

While the proposed generally applicable optimizations could be incorporated into any encoding schemes used for DNA storage, the improvements towards a modified distribution function can only be applied to encoding schemes incorporating fountain codes. As a result, our novel DNA coding scheme developed by incorporating all methods presented in this work, outperforms the existing fountain code schemes DNA Fountain [15], NOREC4DNA [16], and the code presented by Grass et al. [4], regarding all metrics evaluated. For these fountain code-specific optimizations, the results obtained in this work may differ depending on the specific DNA storage approach used.

Biological validation is clearly important to further demonstrate the effectiveness of the presented methods, but our in-silico results indicate the significant improvements achievable. Our optimization methods target measurable metrics such as the number of unique packets, the required overhead for decoding or rule violations, and thus the expected error probability, which are commonly used in the field of DNA data storage to filter or adapt sequences before synthesis. For example, it is well-established that DNA sequences with longer homopolymer runs generally expose a higher risk of errors during synthesis and sequencing compared to sequences with shorter runs [53], [4], [50], [16]. As a consequence, information-theoretic metrics such as entropy can be used to describe the randomness of DNA sequences, which negatively correlates with the probability of homopolymer runs or unbalanced GC content that may lead to errors. Additionally, common biological metrics such as secondary structure prediction using Gibbs free energy calculation described in Section 3.5 highlight that our results most likely directly translate into advantages within actual DNA-based storage systems. Furthermore, several of the optimization goals use purely mathematical metrics that are independent of biological aspects, such as maximizing the number of unique packets that can be generated for a given seed space. It is evident that with more unique packets, the overall possible capacity of the code increases. While actual DNA synthesis and sequencing would be required to demonstrate the benefits in practice, optimizing in-silico metrics that are known to be associated with DNA storage reliability and capacity gives confidence that the obtained results can be transferred to real-world systems. When considering modern electrochemical methods for DNA synthesis, which can be seen as the best currently available candidates for massive parallelization of the costly synthesis process, the use of fountain codes and especially our presented methods further highlight their flexibility. Since these electrochemical methods typically expose different restrictions such as shorter maximum sequence lengths, fountain codes can simply adapt to these restrictions by, e.g., changing the chunk length. With the increased number of usable packets due to seed spacing, payload masking, and the use of an optimized distribution function, the negative impact of using shorter sequences can be minimized without requiring a larger seed. Examining the sequencing coverage required for a successful decoding, the improvements introduced in our work are expected to help lowering the costs. Since all presented methods are capable of reducing the average error, which in turn decreases the probability of reading low quality or defective strands during sequencing, a smaller coverage may be sufficient to allow error-free decoding. By reducing the required overhead, increasing the number of unique packets and additionally increasing the percentage of recoverable chunks in the event of a failure to decode, a smaller coverage not including all synthesized sequences may be sufficient to fully decode a stored file or at least allow the retrieval of large portions of its content.

## 5 Conclusion

We presented several novel methods for optimizing fountain codes to enhance their applicability and performance for DNA data storage.

In particular, we introduced optimization methods such as seed spacing to decouple the seed structure from rule violation likelihood, payload masking to increase entropy and reduce error probability, and a novel approach for storing metadata inside the encoded sequences. Furthermore, we developed methods that improve the fountain code degree distribution to balance encoding efficiency, error correction, and storage density. These methods can be used to address the complex, bursty, and input-dependent nature of errors in DNA data storage.

Through extensive in-silico experiments, we demonstrated that the proposed optimizations lead to improvements in key metrics, such as reducing the number of non-unique packets, decreasing the average decoding overhead, increasing the proportion of rule-abiding packets, and minimizing unrecovered data in cases of unsuccessful decodings. The resulting optimized fountain codes outperformed existing implementations like DNA Fountain, NOREC4DNA, and the code presented by Grass et al. in our evaluations. While biological validation is still needed to demonstrate the benefits in practice, optimizing the biologically independent and measurable in-silico metrics known to correlate with DNA storage reliability and capacity provides confidence that our improvements will translate to real-world DNA storage systems. The presented methods lay the groundwork for harnessing the full potential of fountain codes in DNA data storage applications. Thus, the presented methods are relevant for researchers developing new codes for DNA storage, as well as for biologists who wish to use highly optimized and ready to use coding schemes for DNA storage.

There are several areas for future work. For example, while the presented optimization methods were able to improve the chosen fitness function in a stable manner, other black-box optimization algorithms may be used to create custom distribution functions tailored for DNA data storage. Furthermore, research in the area of information embedding as described in Section 2.6.2.2 may be promising to embed file meta-data or simple database entries to create a structured storage in which basic information can be retrieved without the need for decoding all stored files. This approach may also be used to embed biologically relevant markers to access or modify data stored in DNA. For example, by artificially embedding a Lox site into strategically selected packets, it could be possible to implement a kill-switch by employing Cre recombinase to destroy important sections of stored packets. We also envision an optimization with respect to the avoidance of secondary structures as future work, since this may be a relevant factor for the biological realization of large DNA data storage systems. Moreover, it is interesting to investigate to which extent the presented methods could be applied to non-fountain code-based encodings for DNA data storage or how such codes could be modified to profit from the presented methods by employing additional fountain coding concepts. Finally, it should be investigated if increasing the maximum degree beyond 41 is useful to decrease the error probability without simultaneously increasing the required overhead and computational cost during decoding.

## Inclusion and diversity

We support inclusive, diverse, and equitable conduct of research.

## CRediT authorship contribution statement

**Peter Michael Schwarz:** Writing – review & editing, Writing – original draft, Visualization, Validation, Software, Resources, Methodology, Formal analysis, Data curation, Conceptualization. **Bernd Freisleben:** Writing – review & editing, Supervision, Project administration, Methodology, Funding acquisition.

## Declaration of Competing Interest

The authors declare no competing interests.

## Data Availability

The source code of the created software and the generated results are available at https://github.com/umr-ds/OFC4DNA and https://doi.org/10.5281/zenodo.10604145.

## Appendix A Supplementary material

MMC Additional experiments and evaluations for the presented optimization techniques. MMC
