# Supplementary material for: Optimizing fountain codes for DNA data storage
Source: Comput Struct Biotechnol J. 2024 Oct 26;23:3878–96. doi: 10.1016/j.csbj.2024.10.038 (PMC11570749; doi:10.1016/j.csbj.2024.10.038)
Supplement: MMC — Additional experiments and evaluations for the presented optimization techniques. [file mmc1.pdf]

Supplemental material for: Optimizing fountain codes for DNA data storage

Peter Michael Schwarz[1,*] and Bernd Freisleben[1]

October 25, 2024

Affiliation

[1] Department of Mathematics and Computer Science, University of Marburg, Germany
[*] Corresponding author: Peter Michael Schwarz: schwarzk@informatik.uni-marburg.de

Abstract

Fountain codes, originally developed for reliable multicasting in communication networks, are effectively applied in various data transmission and storage systems. Their recent use in DNA data storage systems has unique challenges, since the DNA storage channel deviates from the traditional Gaussian white noise erasure model considered in communication networks and has several restrictions as well as special properties. Thus, optimizing fountain codes to address these challenges promises to improve their overall usability in DNA data storage systems. In this article, we present several methods for optimizing fountain codes for DNA data storage. Apart from generally applicable optimizations for fountain codes, we propose optimization algorithms to create tailored distribution functions of fountain codes, which is novel in the context of DNA data storage. We evaluate the proposed methods in terms of various metrics related to the DNA storage channel. Our evaluation shows that optimizing fountain codes for DNA data storage can significantly enhance the reliability and capacity of DNA data storage systems. The developed methods represent a step forward in harnessing the full potential of fountain codes for DNA-based data storage applications.The new coding schemes and all developed methods are available under a free and open-source software license.

1 Files

Table 1 contains the properties of the used files in terms of size, entropy, and number of chunks used. The majority of files have a size of less than 1 MB, but the bitmap image of the Marburg university logo is larger. It was split into a larger number of chunks, which increased the set of possible chunks to draw from during the creation of packets. Therefore, the probability of generating non-unique packets is less than for files encoded using less initial chunks. The entropy of the used files covers a wide range. While the file "logo_ mosla_bw.bmp" has the lowest entropy of 0.52611, the compressed file "Dorn.zip" has the highest entropy. Since a higher entropy indicates a more random distribution of the binary (or quaternary in the case of DNA-based entropy) values, a file with a high entropy should yield less rule-violating packets than a low entropy file encoded using fountain codes. Since combining more chunks reduces the impact of low entropy, this behavior is often associated withpackets with lower degrees. Furthermore, rule-abiding packets generated using a high entropy input, a masked payload, as well as encoded using a higher degree, will most likely produce encoded sequences closer to 50% GC content and a smaller average homopolymer length than files encoded without these methods. Consequently, GC content and homopolymers are indicators for the overall stability of the encoded sequences, and the decoding process is expected to be more reliable since less errors are expected.

**Filename**	**size (kB)**	**#chunks**	**Entropy**	**Entropy$_{GF(2)}$**
logo_mosla_bw.bmp	45	1,138	0.87256	0.52611
Uni_Marburg_Logo.bmp	1,230	31,463	1.26765	1.45598
Uni_Marburg_Siegel_bw.bmp	158	4,029	2.65599	1.77103
Dorn	5	122	4.23,460	1.91665
logo.jpg	71	1,800	7.22542	1.96631
Dorn.zip	54	1,359	7.99562	1.99996
sleeping_beauty	7	167	4.36472	1.94101
aes_Dorn	5	122	7.96065	1.99977
0010-bmp.bmp	3,015,990	131,130	7.87348	1.99527
0011-bmp.bmp	3,015,990	131,130	7.65862	1.98903
0012-bmp.bmp	3,015,990	131,130	7.12314	1.87614
0013-bmp.bmp	3,015,990	131,130	7.73894	1.98856
0014-bmp.bmp	2,289,846	99,559	7.50563	1.98777
0020-xlsx.xlsx	1,153,561	50,155	7.96420	1.99852
0021-xlsx.xlsx	1,190,867	51,777	7.96554	1.99877
0022-xlsx.xlsx	129,709	5,640	7.66694	1.99186
0023-xlsx.xlsx	13,273	578	7.25740	1.94210
0024-xlsx.xlsx	14,962	651	7.35421	1.94671
0030-zip-highcompress.zip	160,369	6,973	7.99787	1.99986
0031-zip-highcompress.zip	2,803,208	121,879	7.99864	1.99982
0032-zip-highcompress.zip	4,835,094	210,222	7.99939	1.99993
0033-zip-highcompress.zip	362,497	15,761	7.99878	2.00000
0034-zip-highcompress.zip	2,159,548	93,894	7.99969	1.99996
0040-txt.txt	431	19	4.84797	1.93405
0041-txt.txt	256	12	5.02297	1.93715
0042-txt.txt	2,153	94	5.01285	1.93529
0043-txt.txt	1,582	69	4.63744	1.96039
0044-txt.txt	623	28	4.64018	1.93573

Supplemental Table 1: Size, number of chunks, and computed entropy for the different files used in this experiment. The numbered entries are taken from the "NapierOne" dataset.

2 Restrictions

The following restrictions were used to determine the error value of a sequence:

1. Maximum Homopoylmer length ≤ 4
2. Global GC content: 40 < GC < 60
3. Windowed GC content: 40 < GC < 60 for a window size of 50
4. No use of undesired subsequences as defined in Table 2

Lox sites	ATAACTTCGTATAGCATACATTATACGAAGTTAT
	ATAACTTCGTATAGCATACATTATACGAACGGTA
	TACCGTTCGTATAGCATACATTATACGAAGTTAT
	TACCGTTCGTATAGCATACATTATACGAACGGTA
	TACCGTTCGTATATGGTATTATATACGAAGTTAT
	TACCGTTCGTATATTCTATCTTATACGAAGTTAT
	TACCGTTCGTATAGGATACTTTATACGAAGTTAT
	TACCGTTCGTATATACTATACTATACGAAGTTAT
	TACCGTTCGTATACTATAGCCTATACGAAGTTAT
	ATAACTTCGTATATGGTATTATATACGAACGGTA
	ATAACTTCGTATAGTATACCTTATACGAAGTTAT
Twister 5' Adapter	GAAGTGCCATTCCGCCTGACCT
Twister 3' Adapter	AGGCTAGGTGGAGGCTCAGTG

Supplemental Table 2: Undesired DNA subsequences for all experiments. The presence of the listed subsequences increases the error value of the sequence to 100%.

3 Pair Plots

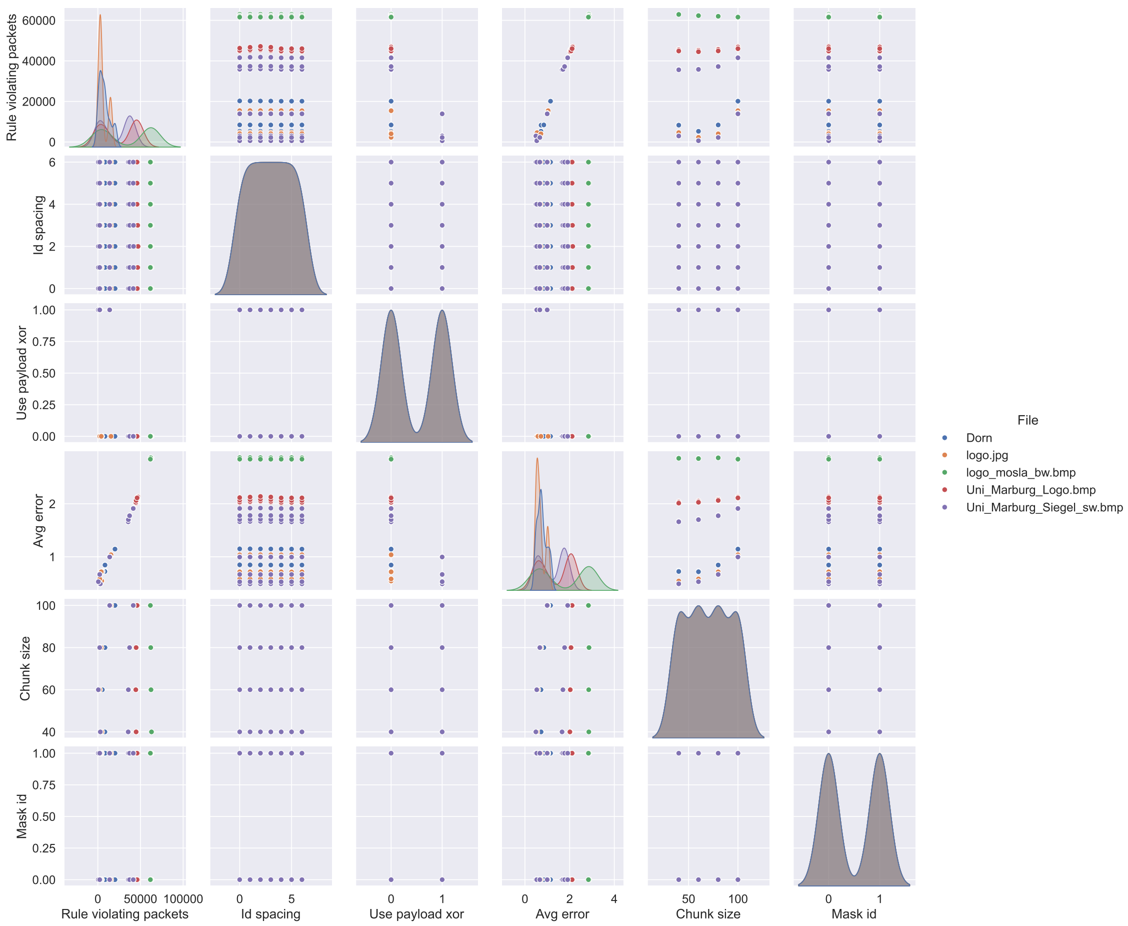

Supplemental Figure 1: Pair plot for all tested metrics given different input files

The pair plot shown in Figure 1 illustrates the impact of the files used in the comparison. Most importantly, the impact of the seed spacing on the number of rule-violating packets can be observed, as well as the influence of the chosen chunk size on both the average error and the rule-violating packets. Additionally, the distribution of the average error and the number of rule-violating packets confirm that the information rates of the input files have a significant impact on these metrics.

Figure 2 indicates that there is a significant impact of increasing chunk sizes on the average error and the number of rule-violating packets. A less significant observation is evident for the seed spacing and the seed masking setting. For the seed masking option, no directly visible improvement can be seen, whereas the seed spacing parameter indicates a consistent influence on both performance metrics displayed. These observations could have been expected by the previous evaluations, but the pair plot additionally

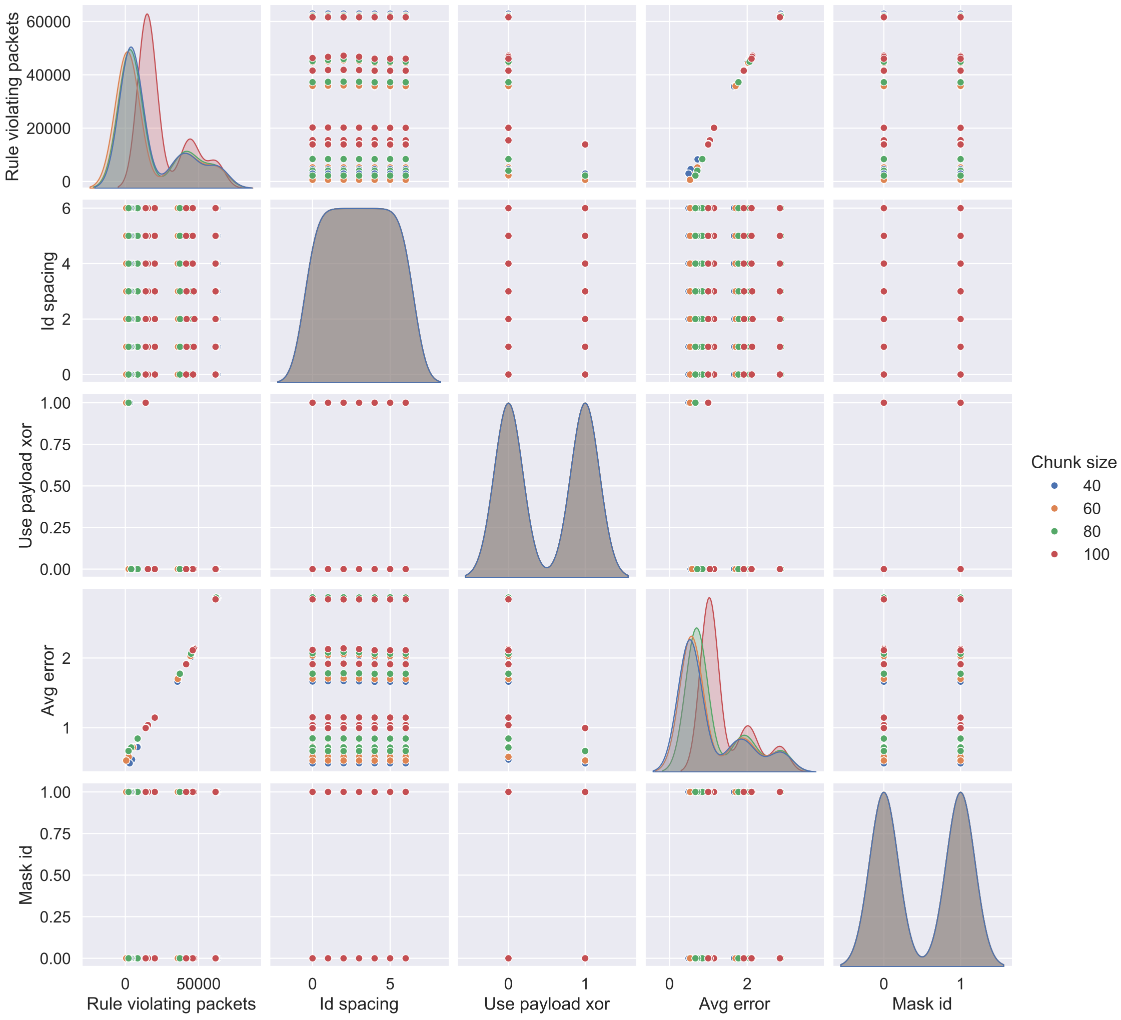

Supplemental Figure 2: Pair plot for all tested metrics given different chunk sizes

shows that the use of the payload XOR method significantly reduces the expected number of rule-violating packets and thus the average error rate.

This behavior is further confirmed in Figure 3. In this pair plot, the use of the payload XOR option is analyzed. This option has a significant impact on all observed metrics.

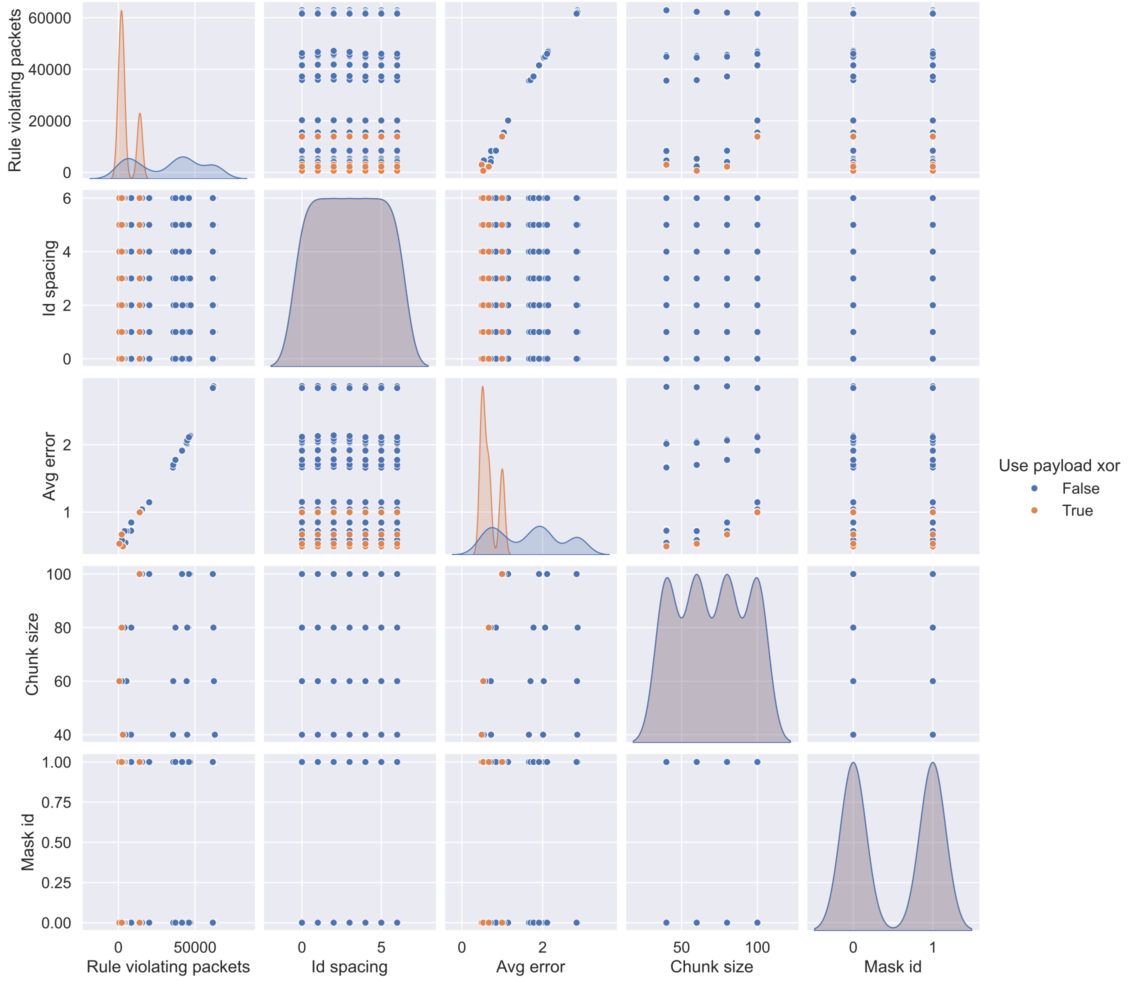

Supplemental Figure 3: Pair plot for all tested metrics when employing payload masking with the XOR method

4 Seed masking

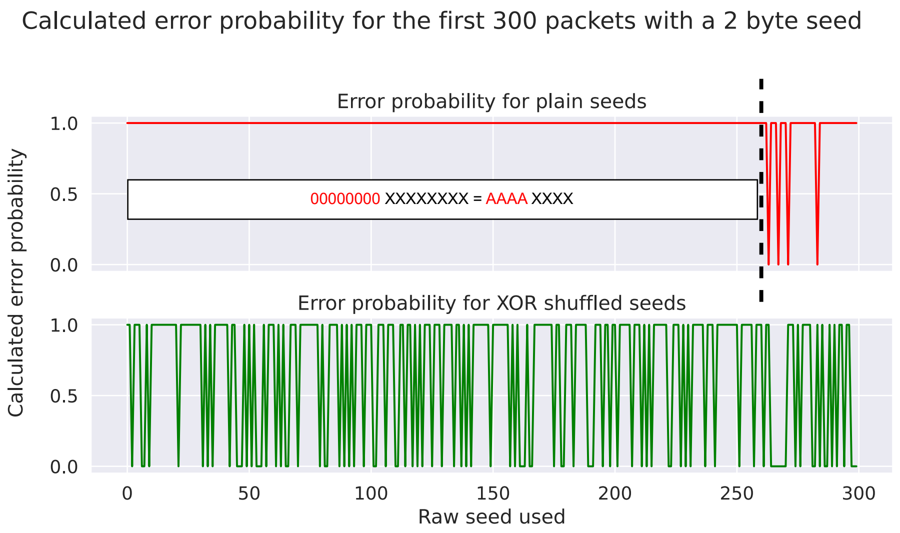

Supplemental Figure 4: Seed masking: Distribution of the first 300 created packets. Without employing seed masking with a fixed XOR-mask, the first 256 packets have an error probability of 100% due to the presence of homopolymers at the beginning of the seed. This effect becomes even more significant when using a 4-byte seed. The overall number of erroneous packets does not (significantly) change.

Figure 4 illustrates the impact of applying an XOR mask to each seed. In the experiment using the raw seed (depicted in red), the first 256 created packets produce an error probability of 100%, mainly due to the presence of seeds leading to homopolymers. In contrast, the experiment with the XOR-masked seed (depicted in green) distributes the packets that would contain homopolymer-producing seeds across the entire seed space. Furthermore, the behavior of the plain IDs shown in Figure 4 is repeated for each range of seeds that would produce a homopolymer (e.g., 01010101 . . . , 11111111 . . . , 10101010 . . . , . . .).

5 Payload masking

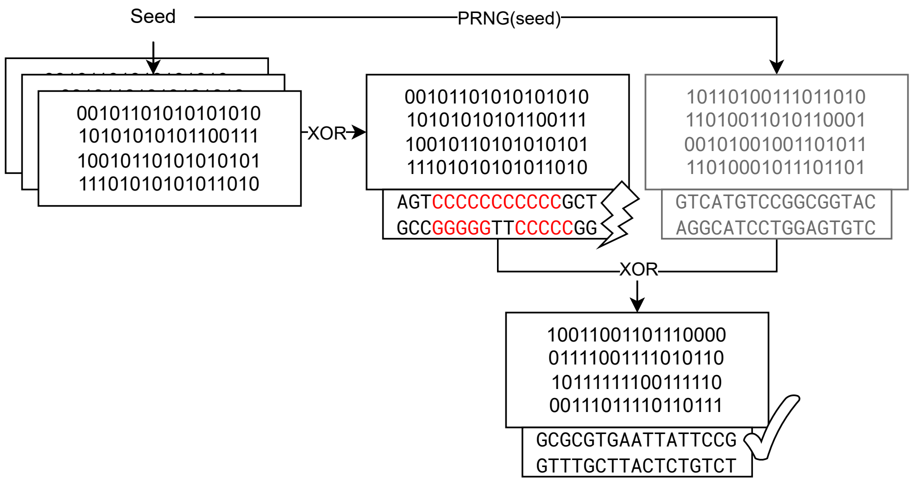

Supplemental Figure 5: Payload masking using a random sequence generated from the seed. The initial sequence produces multiple homopolymers longer than 4. However, by adding (using XOR) a uniformly random sequence of the same length, the final sequence is free of any homopolymers.

6 Seed spacing

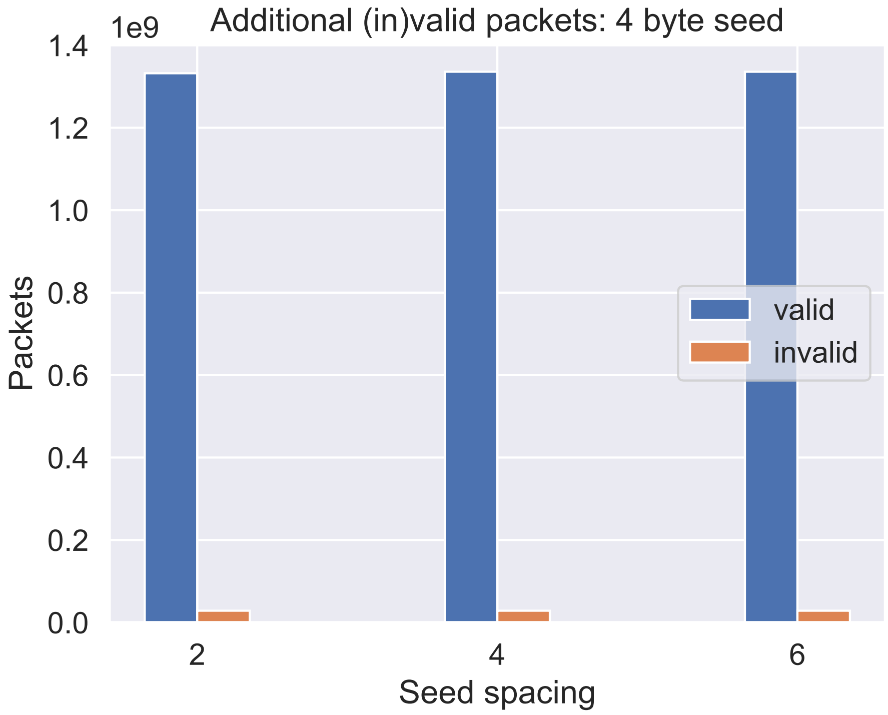

Supplemental Figure 6: Effect of seed spacing compared to the baseline without any seed spacing when restricting valid packets to a maximum homopolymer run of 2 and a GC content between 40% and 60%.

7 Distribution optimization

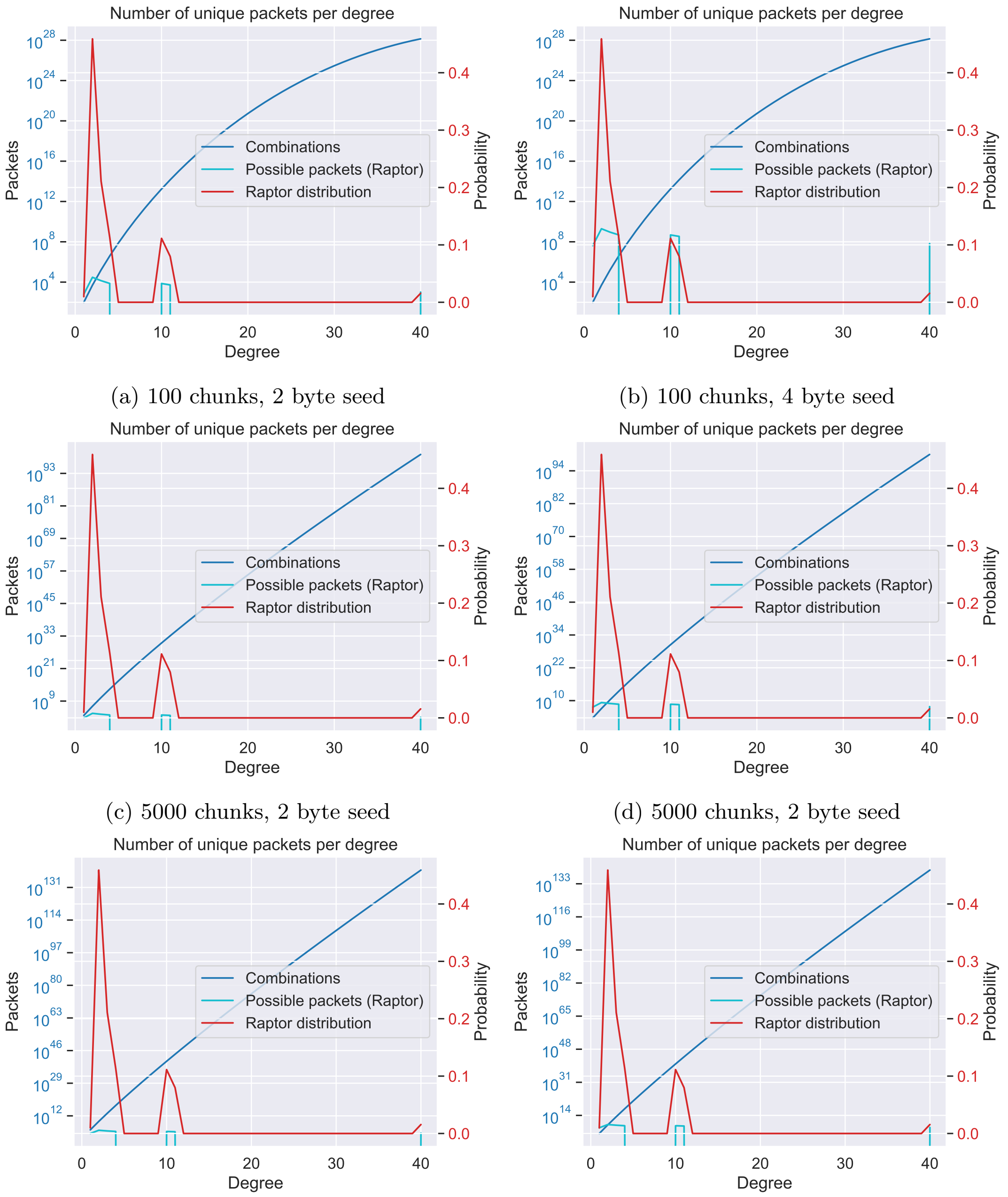

(a) 100 chunks, 2 byte seed

(b) 100 chunks, 4 byte seed

(c) 5000 chunks, 2 byte seed

(d) 5000 chunks, 2 byte seed

(e) 50000 chunks, 2 byte seed

(f) 50000 chunks, 4 byte seed

Supplemental Figure 7: Possible combinations for different number of chunks and seed spaces

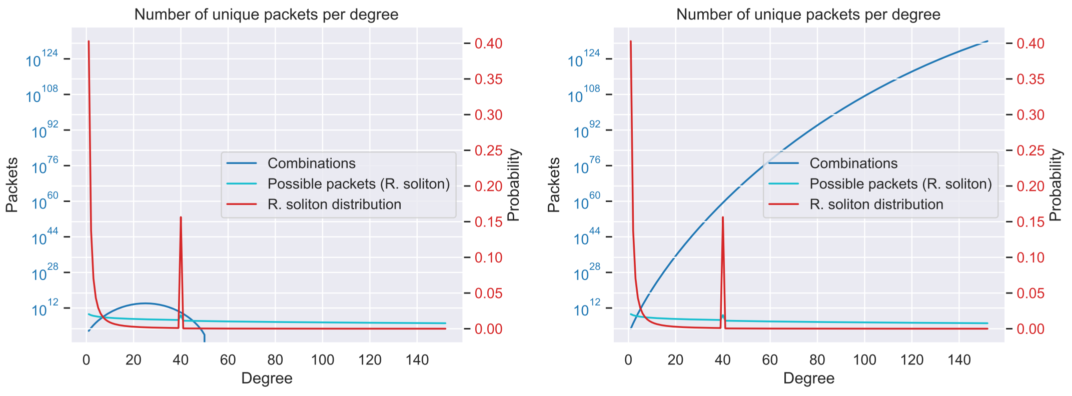

(a) 50 chunks, 4 byte seed

(b) 500 chunks, 4 byte seed

Supplemental Figure 8: The robust soliton distribution function as used in DNA fountain [1] (red), the maximal possible combinations for each degree (blue), as well as the number of possible packets based on this distribution (light blue) for various numbers of chunks and seed sizes.

Figure 8 shows the same experiment for the robust soliton distribution using the default parameters defined in the implementation of Erlich and Zielinski [1]. While this distribution theoretically allows packets to have a considerably higher degree, it heavily favours a small number of low degrees.

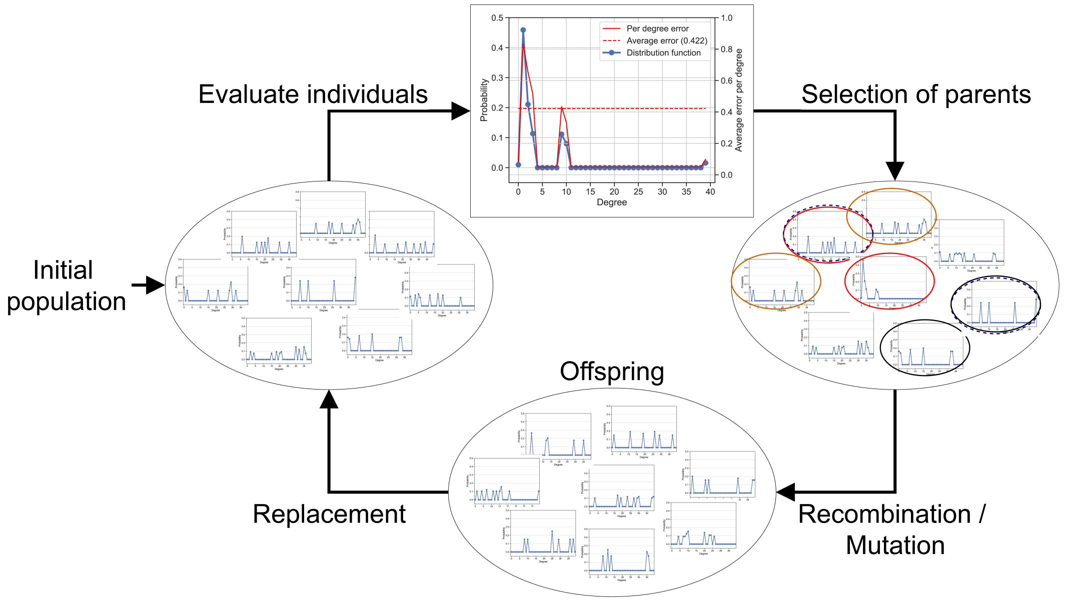

Supplemental Figure 9: Illustration of evolutionary optimization. Evolutionary algorithms optimize a population of individuals using mechanisms inspired by biological evolution.

As outlined in Figure 9, evolutionary algorithms imitate biological evolution by using a fitness function to select and recombine the most suitable individuals in a population. By using advanced recombination and mutation operators as well as a selective replacement of individuals in the population, any pointwise computable functions can be optimized.

The optimization methods used in this work can be described using the following pseudocode:

Examining the number of rule-conforming sequences in Figure 10, we observe an initial improvement followed by a modest but consistent improvement. Since the probability of rule violations is correlated with the entropy of the produced packets, which, in turn, relates to the entropy of the input and the degree used during creation, substantial improvements were not expected.

Figures 12 compares the custom distribution functions using all $5,000$ sequences generated and payload masking enabled. While all variants expose a stable distribution centering around -30, the experiment using differential evolution optimized for high entropy input and a seed spacing of 2 exposes a slighlty better mean value.

Figure 14 further indicates that for files with a lower entropy, such as the BMP and ZIP files, compared to the baseline encoding and the DNA Fountain approach, the optimized versions can produce a mean GC content of almost exactly 50% with a significantly smaller spread into both directions. Focusing on

Algorithm 1 Evolutionary Algorithm

Require: Population size NP, mutation rate F, exit condition EC
Initialize population P with N individuals ▷ P random distribution function
Evaluate fitness of each individual in P ▷ encode all packets for all configurations for each distribution
while not EC **do**
  for $i = 1$ to NP **do**
    Select parents from P based on fitness ▷ select two individuals from P using weighted probability draws
    Merge parents to produce offspring ▷ uses per-degree average error, overall average error, boundaries and normalization
    Apply mutation to offspring with mutation rate F
  end for
  Evaluate fitness of offspring
end while
return best individual in the final population

Algorithm 2 Differential Evolution

Require: Population size NP, mutation rate F, crossover rate CR, exit condition EC
Initialize population P with N individuals ▷ P random distribution function
Evaluate fitness of each individual in P ▷ encode all packets for all configurations for each distribution
while not EC **do**
  for $i = 1$ to NP **do**
    Select three distinct individuals from P: $\mathbf{y}_1, \mathbf{y}_2, \mathbf{y}_3$
    Generate mutant vector $\mathbf{v}_i = \mathbf{y}_1 + F \cdot (\mathbf{y}_2 - \mathbf{y}_3)$ ▷ Mutation
    Choose r as a random degree from the vector $\mathbf{v}_i$
    Generate trial vector $\mathbf{u}_i$ by crossover between $\mathbf{x}_i$ and $\mathbf{v}_i$ based on CR ▷ Crossover each degree based on CR or if degree is equal to r ($\mathbf{x}_i \in P$), ensure boundaries and normalization
    if $f(\mathbf{u}_i) \leq f(\mathbf{x}_i)$ **then** ▷ f is the fitness function
      $\mathbf{x}_i \leftarrow \mathbf{u}_i$ ▷ Selection
    end if
  end for
end while
return best individual in the final population

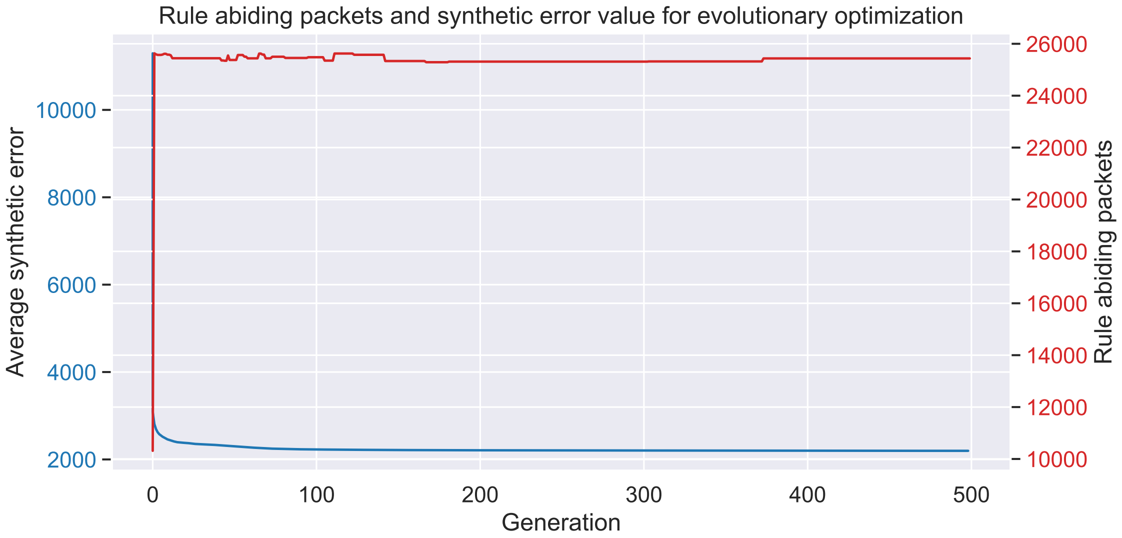

Supplemental Figure 10: Results of the evolutionary optimization algorithm for a high-entropy input: After an initial significant improvement, optimizing the distribution function for 500 generations did not significantly change the number of rule-conforming packets.

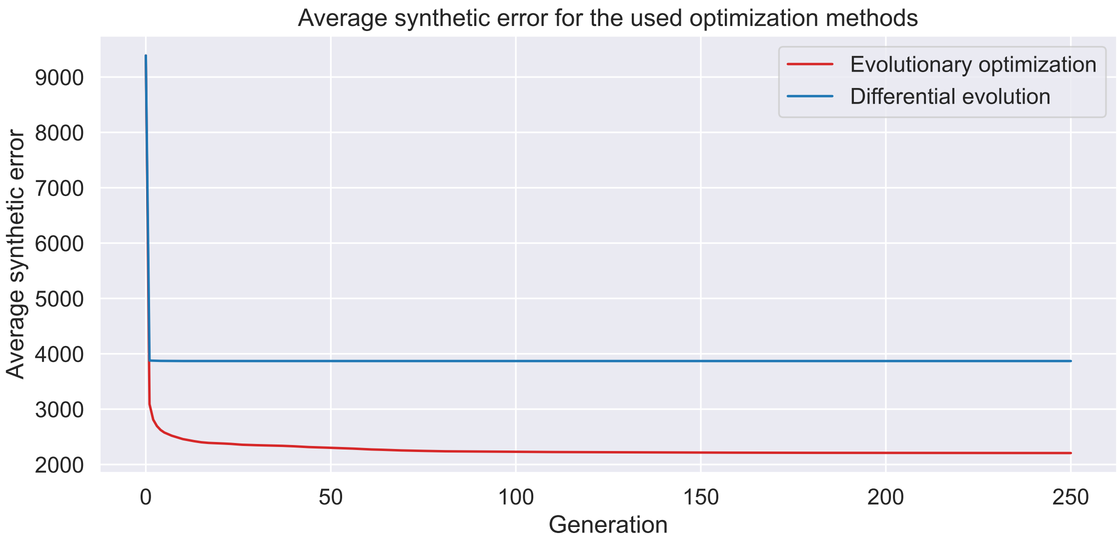

Supplemental Figure 11: Comparison of evolutionary optimization and differential evolution for the AES-encrypted text file using both seed spacing and payload masking. For the evolutionary algorithm, the mutation rate was 0.2 with a population size of 100. For differential evolution, a crossover rate of 0.8 and $f = 0.8$ was chosen.

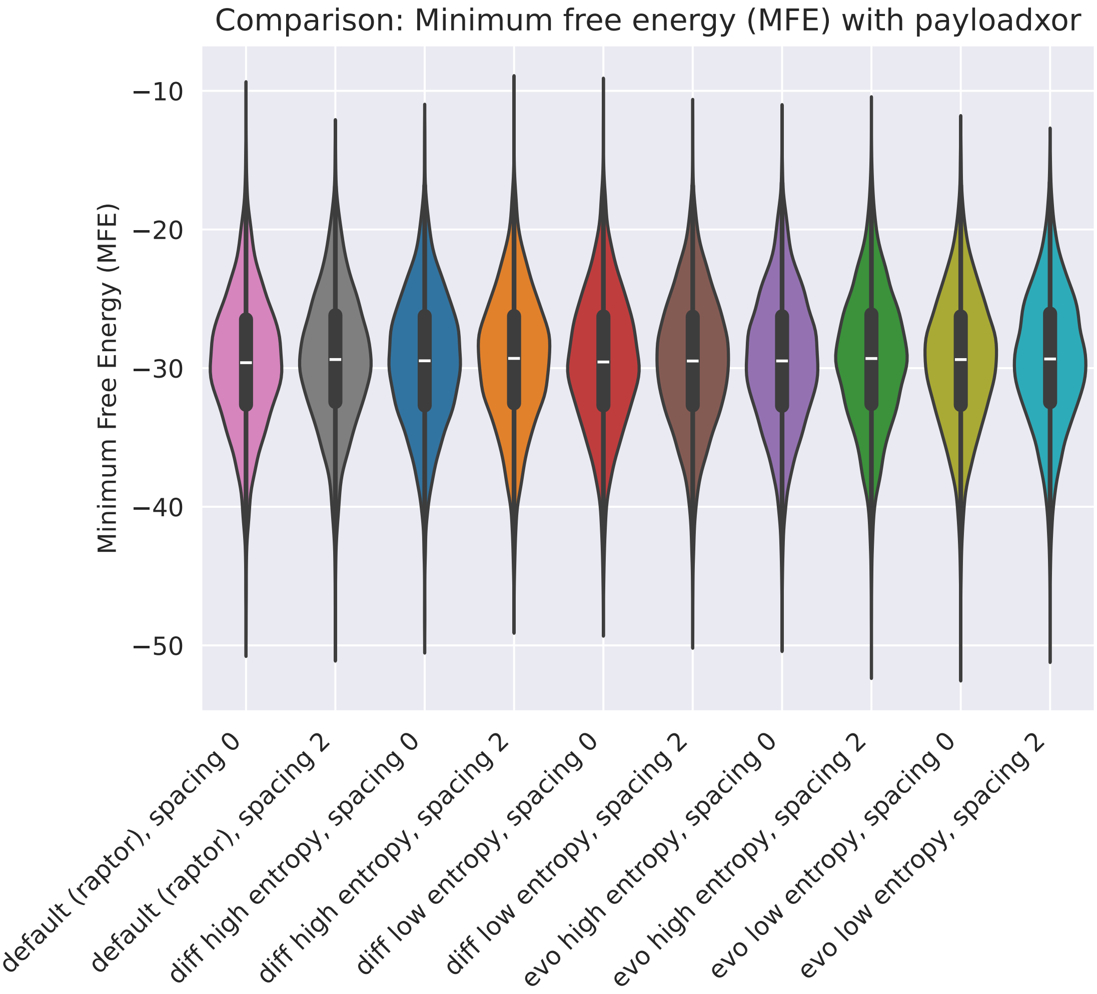

Supplemental Figure 12: Minimum free energy for the *Sleeping Beauty* file for the various distribution functions using payload masking. All experiments contain 5,000 sequences.

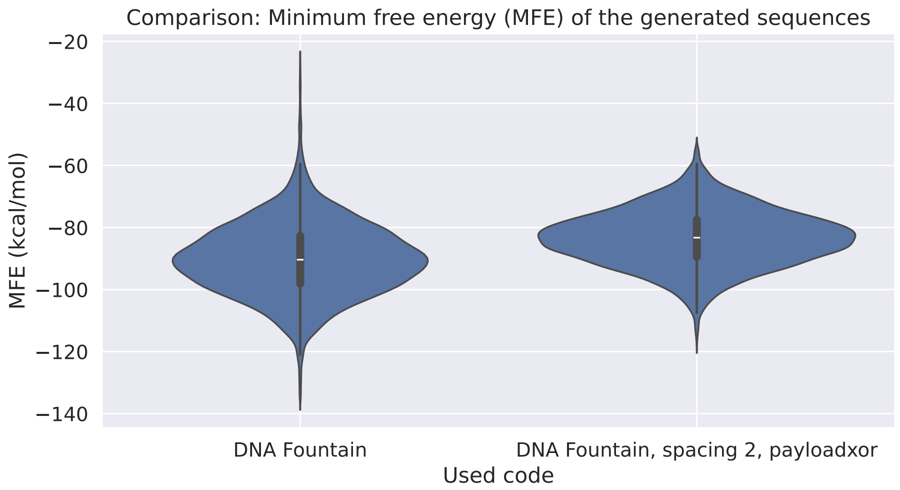

Supplemental Figure 13: Minimum free energy for the *Sleeping Beauty* file encoded into 5,000 sequences of length 300 nt

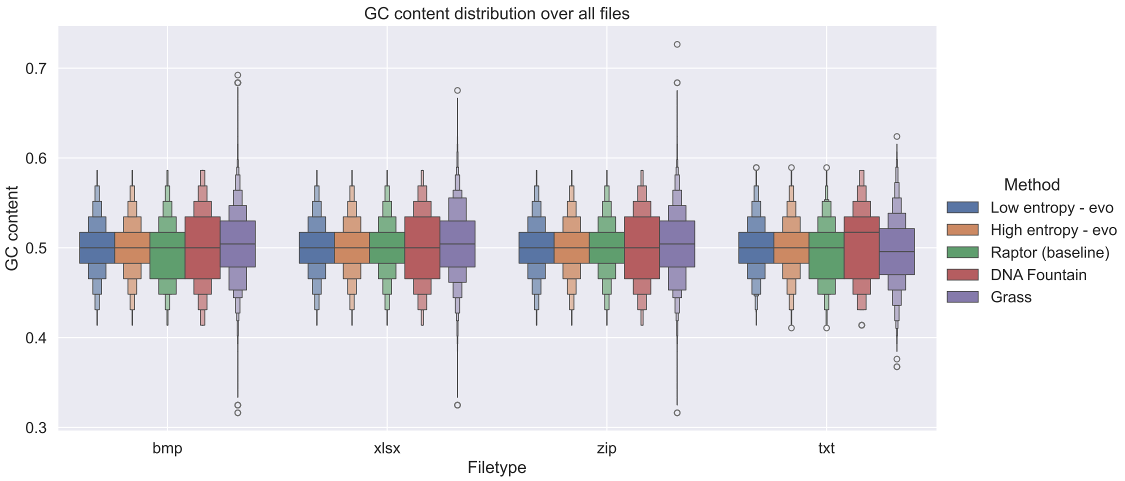

Supplemental Figure 14: Distribution of the GC content for valid packets generated using the different encoding methods

the TXT group, it is evident that the distribution optimized for low entropy files slightly outperforms the version optimized for high entropy data. In contrast, for the high entropy file types XLSX and ZIP, no significant improvements compared to the baseline version are observed.

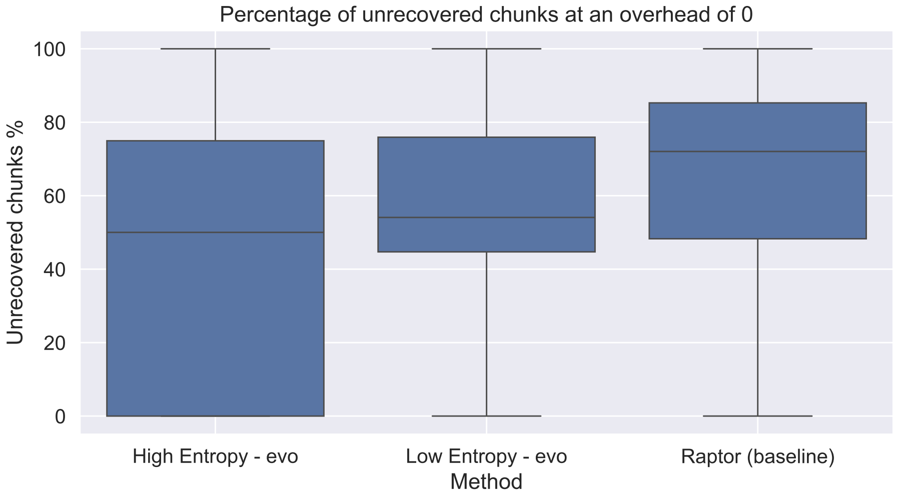

Supplemental Figure 15: Percentage of unrecovered chunks when decoding using a random subset of packets without any overhead

**Impact Factor**	**Value**
f_{overhead}	0.2
$f_{\text{avg\_err}}$	0.4
$f_{\text{clean\_avg\_err}}$	0.2
$f_{\text{non\_unique\_packets}}$	0.3
$f_{\text{unrecovered\_packets}}$	0.1

Supplemental Table 3: Impact factors used in the fitness function

Table 3 shows the impact factors used during the optimizations. These factors were determined based on an iterative process of experimentation and analysis. Furthermore, the choice was made to reflect the special restrictions of DNA as a storage medium, which is currently defined by high costs, a complex set of restrictions, and the inability to produce additional packets compared to a data transmission channel. Therefore, the main focus was the reduction of the number of non-unique packets and the average error, since this directly impacts the maximum file size that can be stored for any given seed range, increases the set of usable packets, and decreases the probability of typical errors present in DNA data storage.

7.1 Optimization for low entropy input

To test the effectiveness of the implemented optimization methods, in the following results, no seed spacing or payload masking was used.

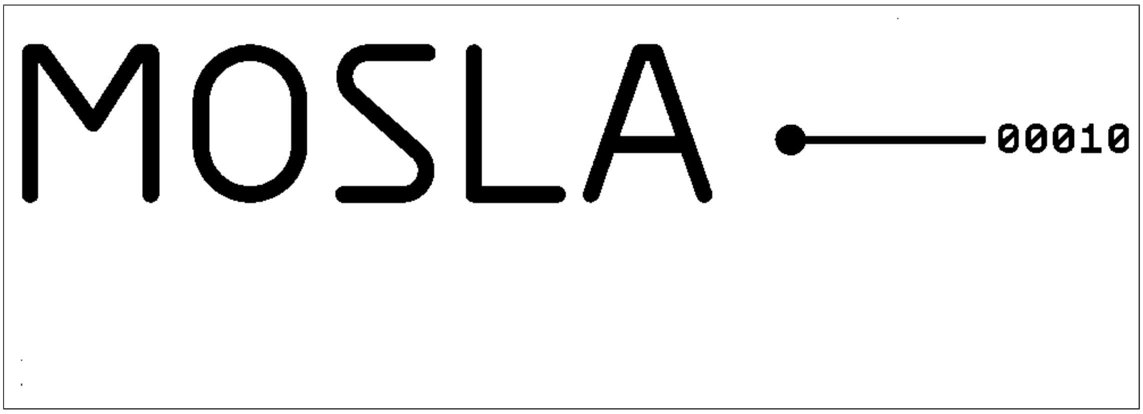

Supplemental Figure 16: logo_mosla_bw.bmp: Low entropy bitmap image used to optimize a fine-tuned distribution function.

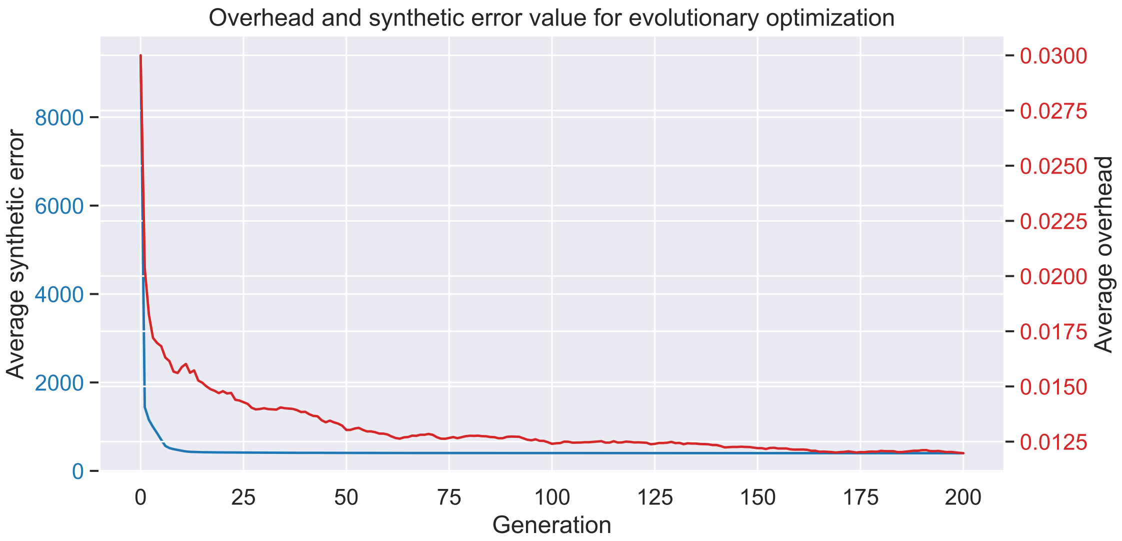

Supplemental Figure 17: Average synthetic error and average required overhead for a low entropy BMP input file using evolutionary optimization, demonstrating a steady decline.

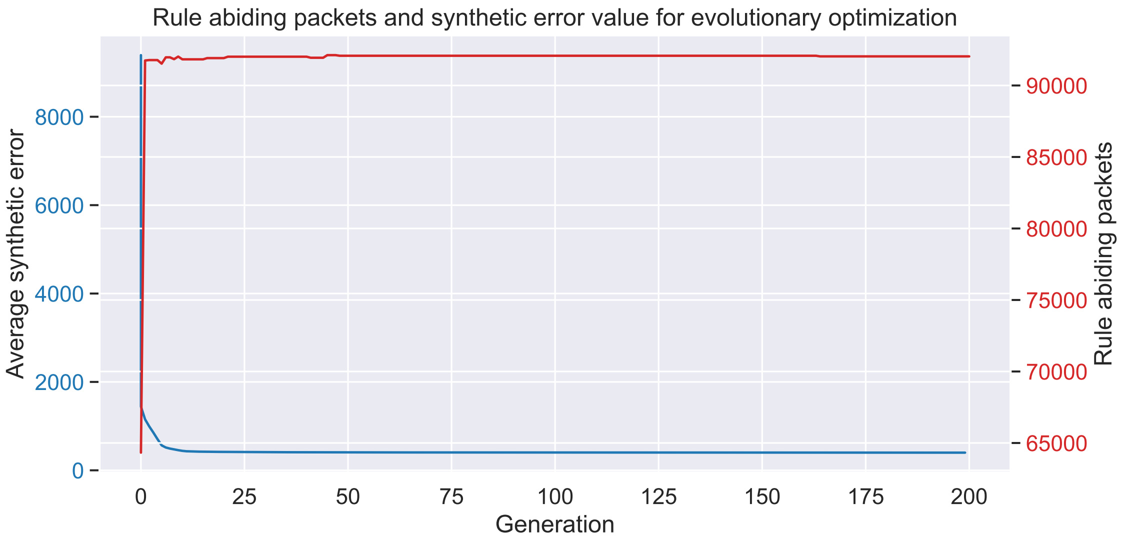

Supplemental Figure 18: Average synthetic error and number of rule-abiding packets for a low entropy BMP input file using evolutionary optimization. Following an initial increase and small fluctuation, the number of rule-conforming packets shows no significant growth.

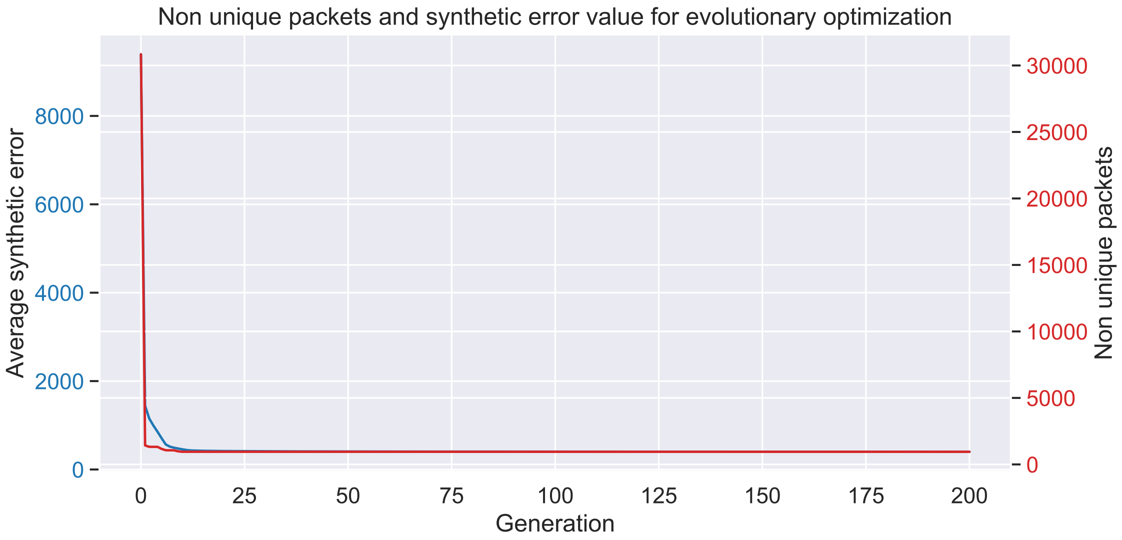

Supplemental Figure 19: Average synthetic error and number of non-unique packets for a low entropy BMP input file using evolutionary optimization, demonstrating the expected strong correlation.

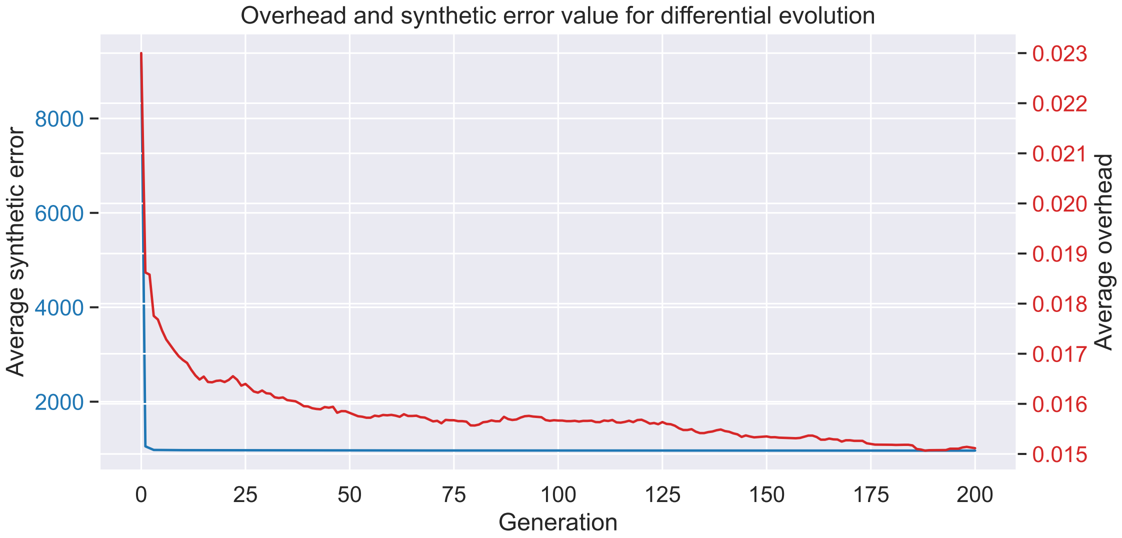

Supplemental Figure 20: Average synthetic error and average required overhead for a low entropy BMP input file using differential evolution, demonstrating a steady decline.

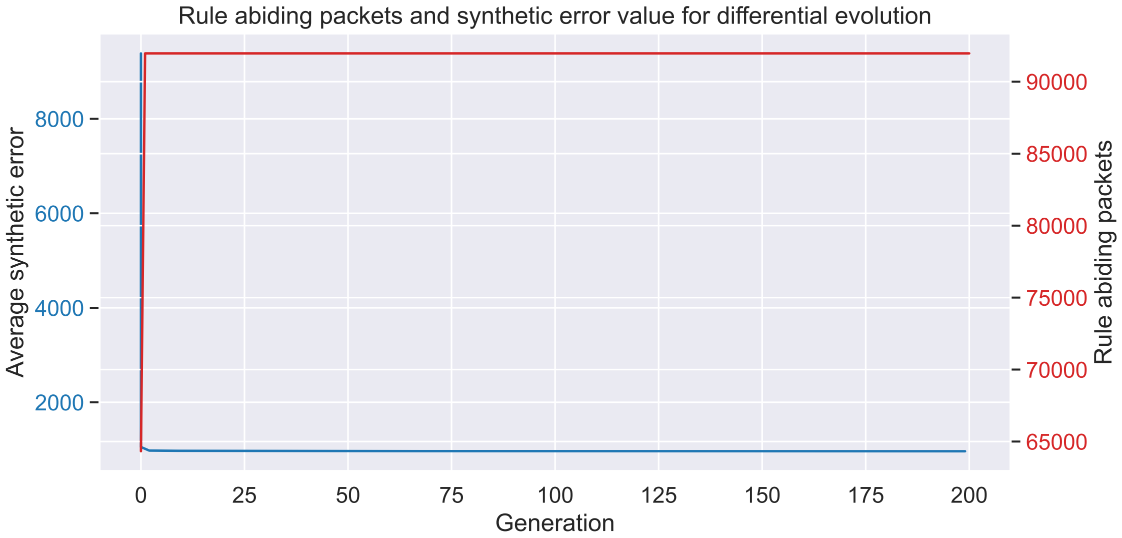

Supplemental Figure 21: Average synthetic error and number of rule-abiding packets for a low entropy BMP input file using differential evolution. Following an initial increase, the number of rule-conforming packets shows no significant change.

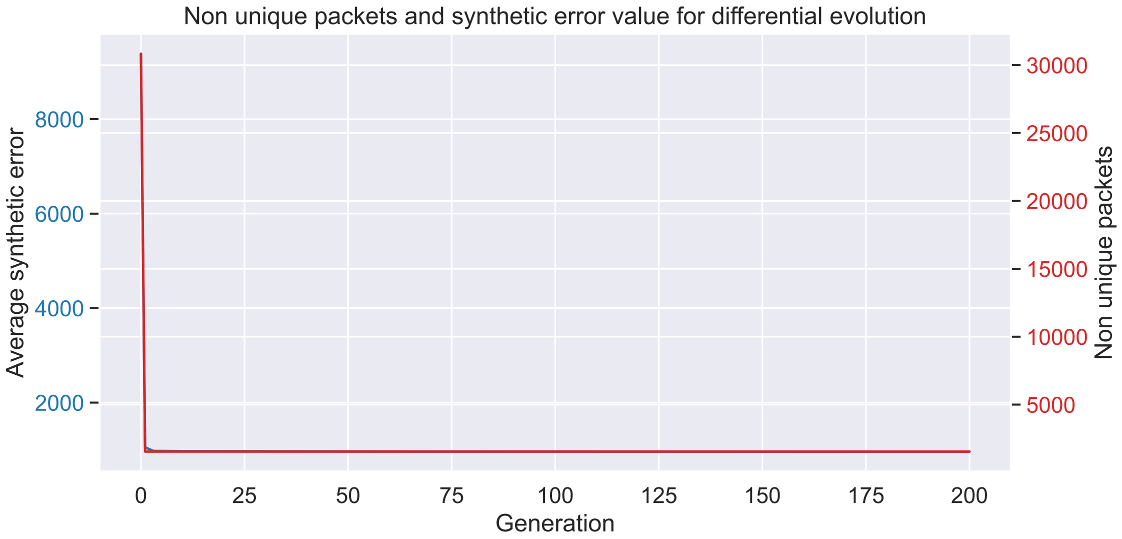

Supplemental Figure 22: Average synthetic error and number of non-unique packets for a low entropy BMP input file using differential evolution, demonstrating the expected strong correlation.

8 Hyperparameter comparison

For all shown figures, the AES-encrypted *Sleeping Beauty* novel was encoded using a chunk size of $40, 60$ and 80 as well as the following optimization factors: $f_{overhead} = 0.2$, f_{avg_error}, $f_{clean\_deg\_len} = -0.0001$, $f_{clean\_avg\_error} = 0.2$, $f_{non\_unique\_packets} = 0.3$, $f_{unrecovered\_chunks} = 0.1$, as described in the manuscript.

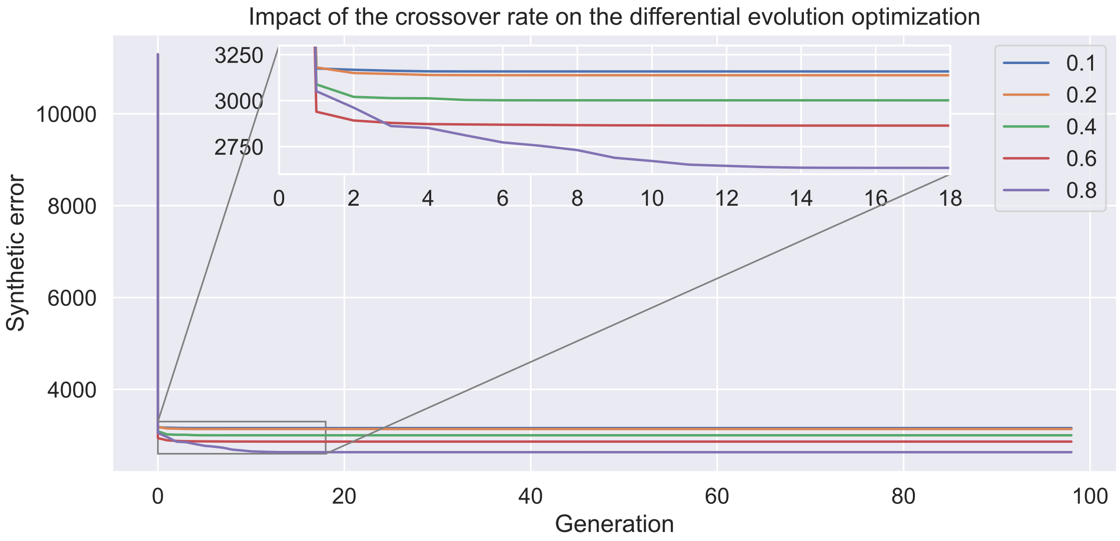

Supplemental Figure 23: Comparison of various crossover rate (cr) values for the differential evolution with a fixed value of $f = 0.8$.

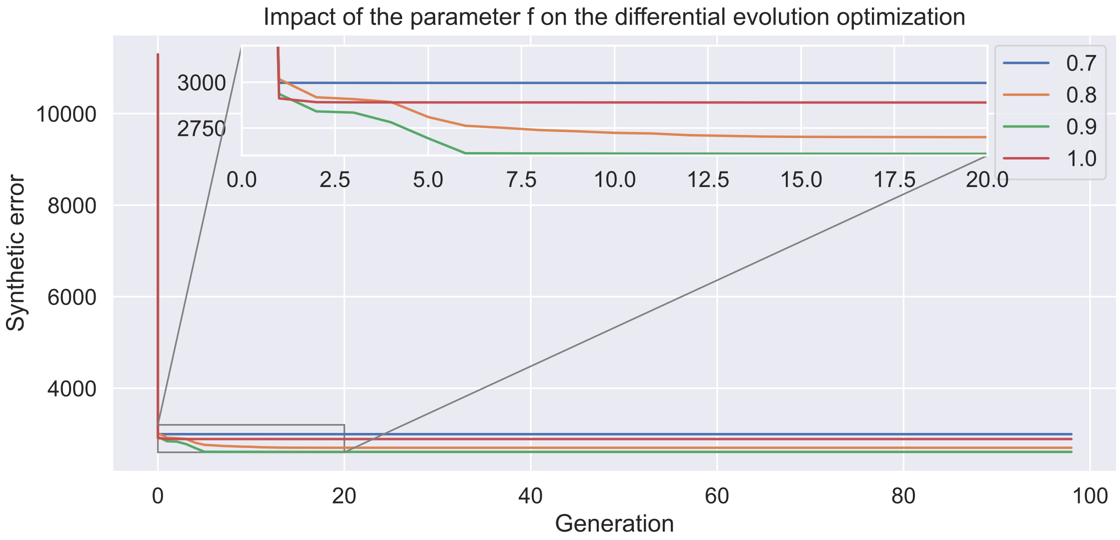

Supplemental Figure 24: Comparison of various values for the hyperparameter f in differential evolution with a fixed crossover rate of $cr = 0.8$.

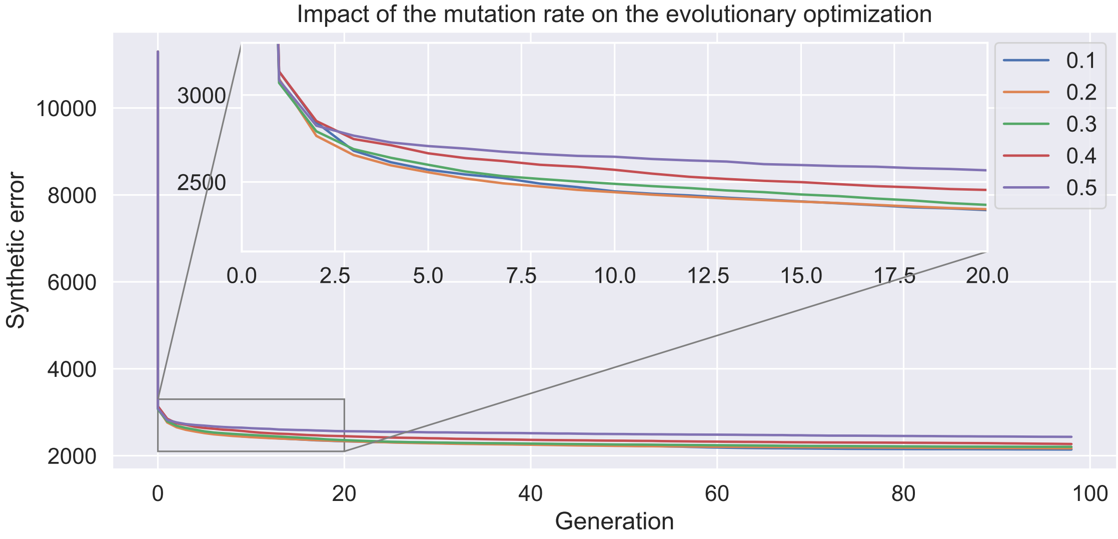

Supplemental Figure 25: Comparison of different values for the mutation rate (mut) for the evolutionary optimization.

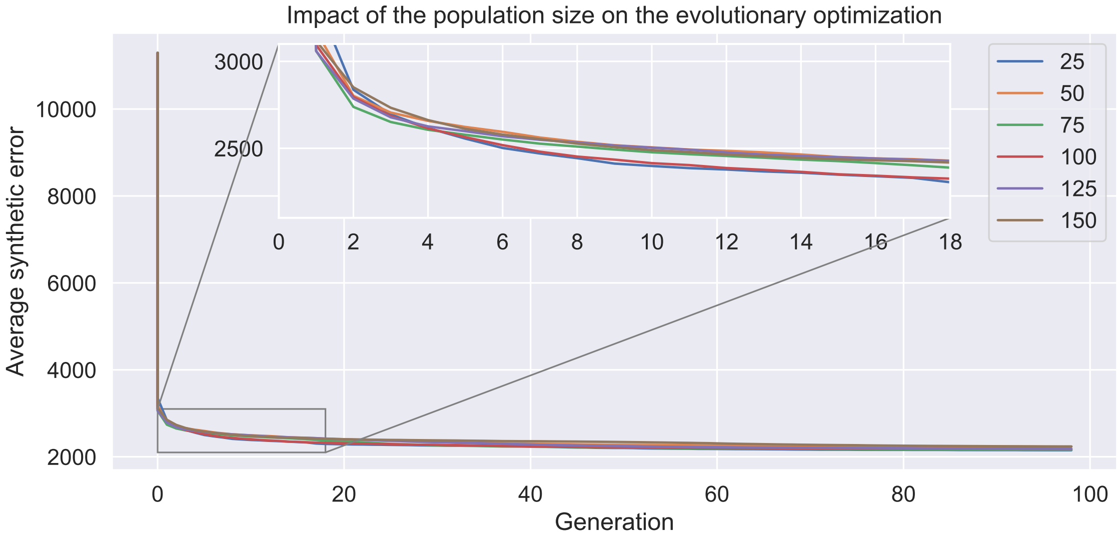

Supplemental Figure 26: Comparison of various values for the population size (pop) for the evolutionary optimization.

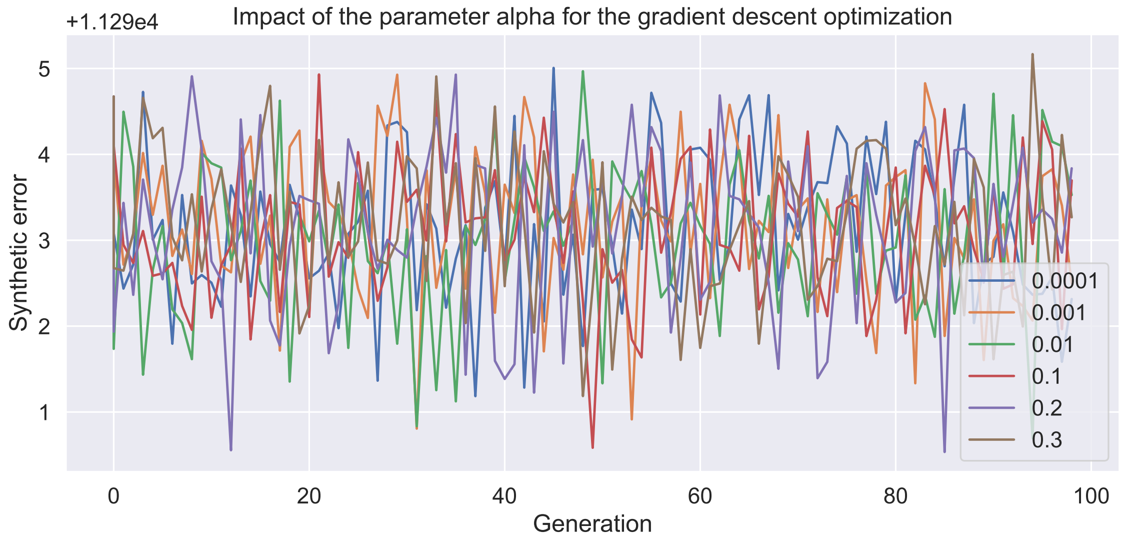

Supplemental Figure 27: Comparison of different values for the population size ($alpha$) for gradient descent. None of the hyperparameters stabilized the optimization for the given high-entropy input.

References

[1] Yaniv Erlich and Dina Zielinski. Dna fountain enables a robust and efficient storage architecture. *Science*, 355(6328):950–954, 3 2017.
